# Energy harvesting from fuel cell bicycles for home DC grids using soft switched DC–DC converter

**DOI:** 10.1038/s41598-024-65482-7

**Published:** 2024-07-05

**Authors:** S. Ramesh, D. Elangovan

**Affiliations:** 1 School of Electrical Engineering, Vellore Institute of Technology, Vellore, 632014 India; 2 Technology Information Forecasting and Assessment Council & School of Electrical Engineering, Vellore Institute of Technology, Vellore, 632014 India

**Keywords:** Fuel cell vehicles, Last-mile transportation, Energy harvesting, DC grid integration, Dual-phase DC to DC converter, Energy science and technology, Engineering

## Abstract

Fuel cell vehicles (FCVs) are gaining significance due to their potential to reduce greenhouse gas emissions and dependence on fossil fuels. Their efficient fuel cell cycle makes them ideal for last-mile transportation, offering zero emissions and longer range compared to battery electric vehicles. Additionally, the generation of electricity through fuel cell stacks is becoming increasingly popular, providing a clean energy source for various applications. This paper focuses on utilizing the energy from fuel cycle bicycles when it's not in use and feeding it into the home DC grid. To achieve this, a dual-phase DC to DC converter is proposed to boost stack voltage and integrate with the 24 V DC home grid system. The converter design is simulated using the PSIM platform and tested in a hardware-in-the-loop (HIL) environment with real-time simulation capabilities.

## Introduction

Electric vehicles (EVs) have gained significant attention and momentum in recent years as the world grapples with the urgent need to address environmental concerns, reduce greenhouse gas emissions, and transition towards a sustainable future. One of the primary reasons for the growing importance of electric vehicles is their environmental advantages. EVs produce zero tailpipe emissions, significantly reducing air pollution and improving air quality in urban areas^1^. Advances in battery technology, charging infrastructure, and vehicle design are making EVs more attractive, affordable, and practical for consumers^2^. However traditional EVs, while commendable for their reduction in greenhouse gas emissions compared to conventional internal combustion engine vehicles, still pose challenges in terms of their overall sustainability^3^. These challenges stem from the reliance on non-renewable energy sources for electricity generation and the environmental impact of battery production and disposal^4^.

Fuel Cell Vehicles (FCVs) represent promising avenue for sustainable transportation. Unlike traditional battery electric vehicles, FCVs utilize hydrogen as a fuel source, producing electricity through a chemical reaction with oxygen. This technology offers several advantages, including fast refuelling times and long driving ranges, making it a compelling alternative for long-haul transportation and heavy-duty applications^5^. According to a report by Bloomberg NEF (BNEF), global EV sales are projected to reach 54 million units by 2040, comprising over 30% of total vehicle sales. Additionally, FCVs, which utilize hydrogen fuel cells to generate electricity, are gaining traction due to their potential for zero-emission transportation and longer driving ranges compared to battery electric vehicles (BEVs)^2^. The Indian government has recognized the importance of promoting EVs and FCVs through various policy measures and incentives. The National Hydrogen Mission launched by the Indian government underscores its commitment to promoting hydrogen-based technologies for clean transportation and energy storage^7^. India has set ambitious targets for reducing greenhouse gas emissions and increasing the share of renewable energy in its energy mix. Hydrogen, particularly green hydrogen produced from renewable sources through electrolysis, can play a vital role in achieving these goals^6^.

Storing hydrogen for fuel cell-powered bicycles poses safety, weight, and cost challenges. Advanced materials are essential to prevent leaks and explosions due to hydrogen's flammability^29^. Compressed gas tanks, though common, are bulky and impractical^30,31^. Presently, Type 2 or Type 3 cylinders, like the 3 L tank at 300 bars used in the Alpha Bike^32^ by Pragma Industries, are prevalent, yet Type 3's power density issues impact efficiency.To overcome limitations, researchers explore metal hydride storage for higher energy density and safety^33^. Recent advancements include improved hydrogen absorption/desorption kinetics and cycling stability through alloying and catalysts, with promising solutions like metal–organic frameworks^34,35^.

Ensuring fuel cell durability in bicycles is crucial given environmental challenges like temperature fluctuations and mechanical stresses. Researchers develop durable catalyst materials and membrane technology to resist contaminants and manage water, extending fuel cell lifespan^36,37^. Addressing operational challenges, such as varying terrains and rider behavior, requires precise hydrogen flow control and energy management. Advanced control systems utilizing model predictive control (MPC) algorithms and sensor fusion techniques optimize performance in real-time^38,39^, making fuel cell bicycles more practical and reliable for everyday use.

In the context of the Indian automotive market, two and three-wheelers occupy a central position, constituting approximately 80% of the total vehicle population^11^. This segment plays a crucial role in catering to the diverse mobility needs of the Indian population, particularly in urban and semi-urban areas where congestion and pollution are prevalent. Also, the focus on two and three-wheelers as the future of fuel cell vehicles in India is both logical and strategic. The potential of fuel cell technology extends beyond just personal mobility. In a country as diverse and vast as India, last-mile transportation poses a unique set of challenges, particularly in rural areas where conventional infrastructure may be lacking^7^.

The concept of a fuel cell bicycle for rural last-mile transportation holds tremendous potential for addressing mobility challenges in remote areas. These innovative bicycles harness the power of hydrogen fuel cells to generate electricity for propulsion, offering an eco-friendly and efficient mode of transportation. Moreover, the simplicity of operation and maintenance makes fuel cell bicycles accessible to a wide range of users, ensuring that even remote communities can benefit from sustainable and convenient last-mile transportation options^8^.

“The current state of fuel cell bicycles makes them impractical for rural areas with limited infrastructure^40^. The high cost, lack of hydrogen stations, and refueling limitations make them less convenient and potentially more expensive than existing options. However, the future holds possibilities for exploration as Worldwide Hydrogen Mission are going on and India is also not an exception of that. As fuel cell technology matures, the price of the bicycles is expected to decrease, improving affordability^41^. The study conducted in^41^ offers a thorough examination of proton exchange membrane (PEM)-based fuel cells and their suitability for fuel cell vehicles as a substitute for internal combustion engine vehicles in New Zealand cities.

Investigating alternative fuels like ammonia or bio-derived hydrogen could offer more rural-friendly options. Additionally, exploring portable hydrogen generation units powered by renewable sources could provide local refueling options in remote areas^42^. Moreover, investments in advanced storage solutions and local production facilities aim to overcome transportation and storage challenges^7,43^ The Indian government's National Green Hydrogen Mission, with a budget of ₹19,744 crore, addresses these costs through subsidies and financial incentives^44,45^. The mission supports creating green hydrogen hubs and decentralized applications with pilot project funding^43,46^. Public awareness campaigns and pilot projects are essential to demonstrate the benefits and safety of hydrogen technology, fostering acceptance in rural areas^45^. Leveraging these strategies, fuel cell bicycles can become a feasible and sustainable transportation option in rural India, aligning with national goals of energy independence and reduced emissions. However, significant advancements in technology and infrastructure are needed before they can compete with traditional and electric bikes.”

Fuel cell technology is not limited to vehicles and industrial applications. Fuel cell stacks are emerging as a viable and environmentally friendly solution for generating power in residential settings. As fuel cell technology continues to advance, it holds the promise of reshaping how we power our homes, in a cleaner and more sustainable era of residential electricity generation^9^. Feeding the home DC grid through a fuel cell bicycle represents a visionary step toward sustainability. This innovative concept envisions a future where not only can fuel cell bicycles provide eco-friendly transportation but also serve as decentralized power generators for homes, especially in rural regions where infrastructure is limited^10^.

**Figure 1:**
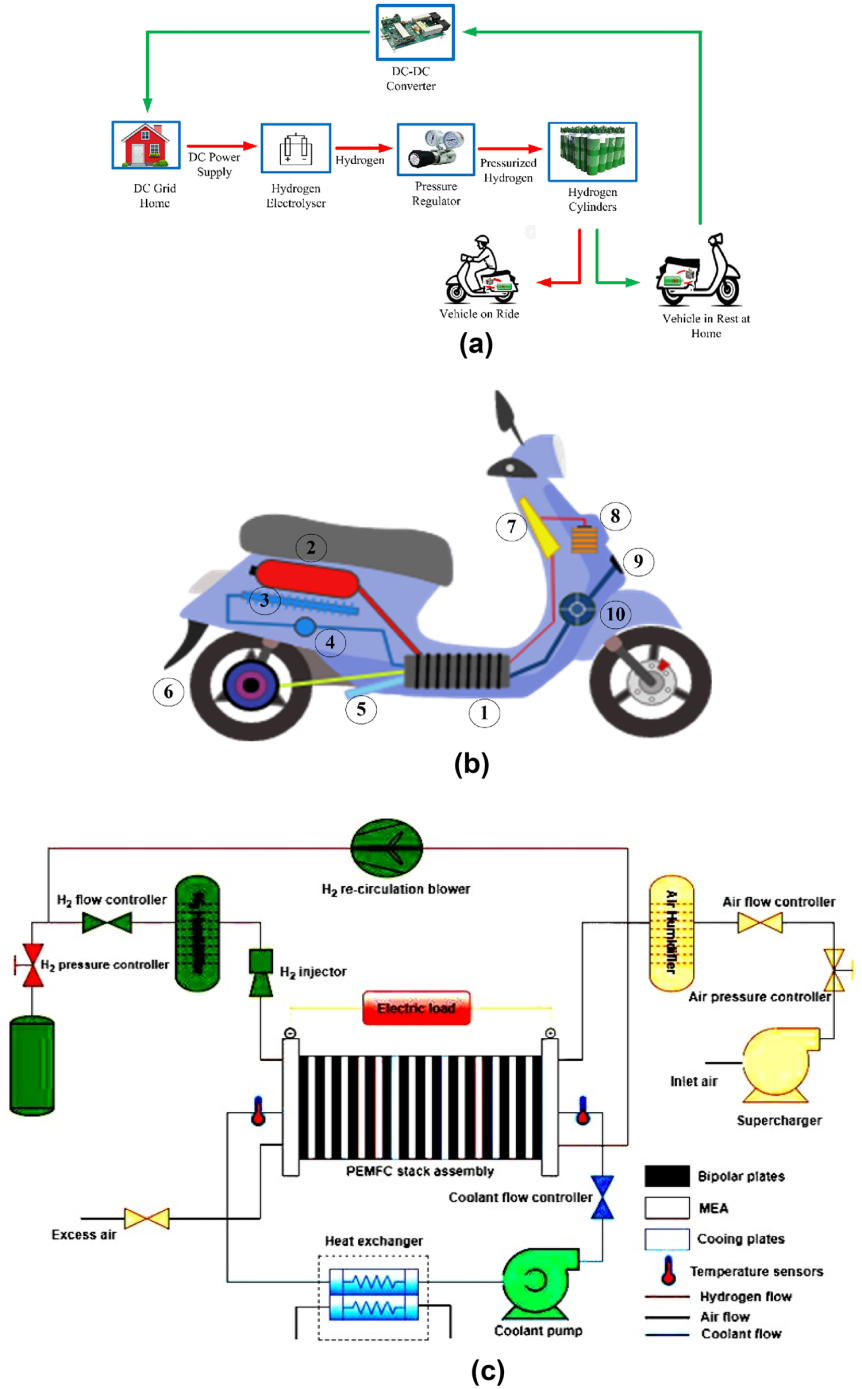
(**a**) Concept of energy transfer in fuel cell powered bicycle. (**b**) Powertrain layout of the fuel cell two-wheeler. 1. PEMFC stack, 2. Hydrogen Cylinder or Metal Hydride Canisters, 3. Heat Exchanger, 4. Water Pump, 5. Water Steam Out, 6. Hub Mounted Motor, 7. Central controller, 8. Auxiliary Battery, 9. Air In, 10. Air Blower. (**c**) general schematic diagram of a hydrogen fuel cell two wheeler.

This paper explores the feasibility and potential of harnessing the energy produced by fuel cell stacks within these bicycles and channelling it into home DC grids using DC–DC Converter as shown in Fig. 1a.

In Fig. 1a the green line indicates when the fuel cell-powered bicycle is not in use, its fuel cell produces electricity for the home's DC grid by using hydrogen from the metal hydride canisters or high pressure tanks through a DC to DC Converter and this innovate system offers numerous advantages. It uses completely renewable energy sources for both household and a transportation need, acts as a self-sufficient microgrid which improves energy access for rural or island communities.

Figure 1b illustrates the powertrain layout of the fuel cell two-wheeler using a traditional powertrain design with metal hydride (MH) canisters for hydrogen storage. This arrangement is also compatible with a gas–solid hybrid cylinder. The hydrogen storage cylinders are situated beneath the seat to facilitate easy replacement of canisters and are thermally linked to the fuel cell stacks through a heat management system.

Figure 1c illustrates a typical schematic of a hydrogen fuel cell two-wheeler. The primary components of this vehicle include hydrogen storage containers, fuel cell stacks, and a vehicle control unit. Differences in fuel cell-powered two-wheelers arise mainly from the variations in hydrogen storage containers and powertrain designs. As a result, the layout of these components and the thermal integration between the hydrogen storage tanks and FC stacks can differ. In cases where hydrogen is stored in metal hydride (MH) canisters, the heat needed for hydrogen desorption is supplied by the cooling water from the FC stacks. This process involves a water pump, a heat exchanger, and a radiator. The vehicle control unit manages the overall operation of the fuel cell two-wheeler.

## DC grid voltage and converter topology selection

On the other hand, utilizing a 24 V DC bus voltage in home grid setup presents numerous benefits, as outlined in various literatures. This voltage level enhances power transmission and distribution efficiency within households by minimizing resistive losses, particularly when compared with lower voltage systems such as 12 V^12^. Although 48 V and 72 V systems may promise superior efficiency, they also pose higher safety risks, especially in residential settings where users may come into contact with the system^13–15^. A 24 V configuration seamlessly integrates with existing home electrical infrastructures, making it a practical choice for retrofitting or upgrading existing setups^15^. It facilitates smooth compatibility with standard appliances and devices. Opting for a 24 V system often proves to be more economically viable compared to higher voltage alternatives like 48 V or 72 V, both in terms of initial installation expenses and ongoing upkeep^16^.

Typically, electric bicycles utilize a 250 W brushless DC motor and for example, Ancheer electric mountain bike uses 250 W brushless geared motor and is designed for both on-road and off-road use. Therefore, in this article, we presume that a fuel cell stack with 250–300 W can power the 250 W bicycle motor.

**Figure 2:**
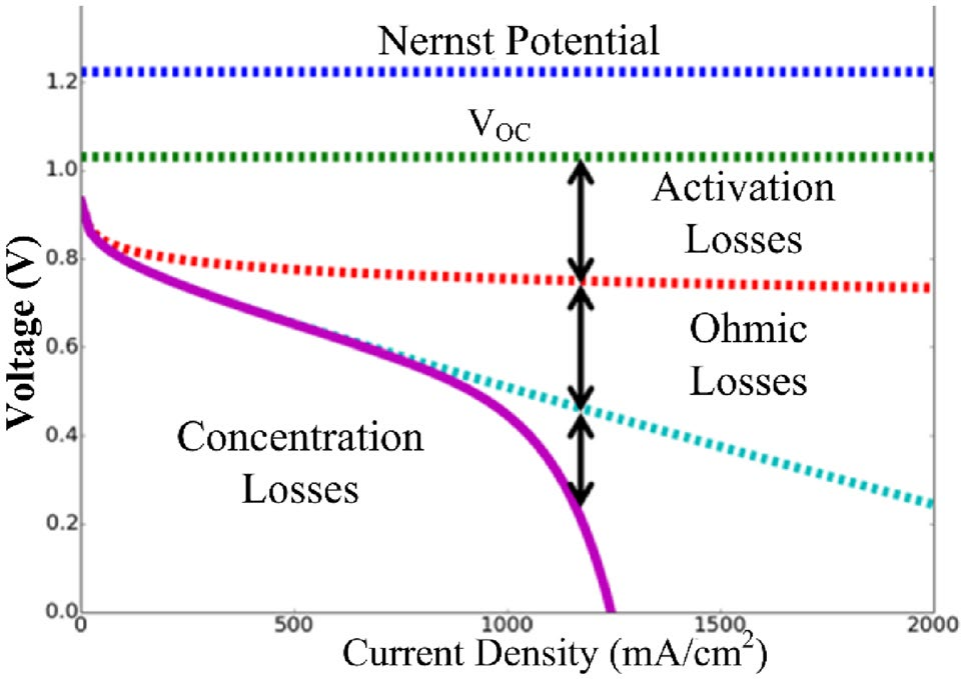
Polarization curve of fuel cell^26^.

Figure 2 shows typical polarization curve of single fuel cell^26^. Theoretically, individual cells have the potential to generate approximately 1.18 V^28^ However, under load conditions, the voltage decreases to approximately 0.6–0.7 V^27^, at this significantly low voltage level, extracting power for mobility or residential usage becomes challenging. Assuming a stack area of 10 * 8 cm^2^ and a single cell stack voltage of 0.6 V, with a current density of 0.7 A/cm^2^, each cell yields a power output of 30–32 W. To meet the energy demands of a 250 W fuel cycle bicycle, 10 cells can be connected in series, practically producing 250–300 W. Then the nominal voltage generated by a 10-cell stack is 6 V.

Hence, we considered a fuel cell stack with 6 V, 250–300 W for driving a bicycle with 250 W Brushless DC (BLDC) motor. However, what sets this system apart is its ability to continue generating electricity even when the bicycle is not in use. The primary focus of this paper is to explore the potential of harnessing power from the 6 V, 250 W fuel cell stack integrated with the bicycle, channelling it to feed a home DC grid with a capacity of 24 V, 250 W using a DC–DC converter.

In the pursuit of DC to DC converters suitable for a 6 V fuel cell stack voltage and a 24 V DC bus voltage at 250W, numerous choices are documented in the literature. Among these, certain combinations prove to be cost-effective and efficient, contingent upon their positioning whether on the low or high side. In a preliminary analysis detailed in^17^, a comparison was made regarding device ratings, turns ratio, device count, and efficiency of half-bridge, current-fed converters, and full-bridge voltage-fed converters on the low side. It was concluded that employing a voltage-fed full-bridge on the low side neither proves cost-effective nor efficient. In another study referenced as^18^, the current-fed converter was positioned on the low side while the voltage-fed converter was positioned on the high side. To maximize efficiency, careful consideration must be given to selecting the active clamp capacitor tailored precisely to the application at hand. However, isolated topology presents notable drawbacks, primarily in terms of component count and cost. With a total of nine active components involved, the increase in cost extends to devices, board space, and control overhead, posing a significant consideration.

The other option is to use non-isolated dc to dc converters. A simple boost converter, coupled inductors topology and other non-isolated converters are available in the literatures. In^19^ author proposed coupled inductor bidirectional dc to dc converter for the fuel cell. To increase the efficiency author uses resonant elements, but come with drawbacks including increased complexity, higher cost, and potential EMI issues. In^20,21^ analysed the boost converter with switched capacitor circuits for fuel cell applications and because of more no of capacitors order of the system increases and the converter response time for dynamic conditions will increase. In^22,23^, the author introduces the dual-level boost and modified dual boost converters, aiming to enhance voltage gain through the incorporation of additional diodes and capacitors. To further amplify the voltage gain, voltage lift switched inductors are employed on the input side of the circuit^24^. However, these converters exhibit a drawback: they require a greater number of components, increasing system costs. Moreover, their reliance on numerous diodes renders them susceptible to reverse recovery losses.

In^25^, the author introduced a modified boost converter aimed at enhancing the efficiency of the conventional boost converter's voltage gain while addressing issues such as output voltage ripple and robustness. However, a drawback of the design was observed: the main diode, directly linked to the input source, incurred on-state losses when the switch was activated, consequently diminishing the converter's efficiency.

**Figure 3:**
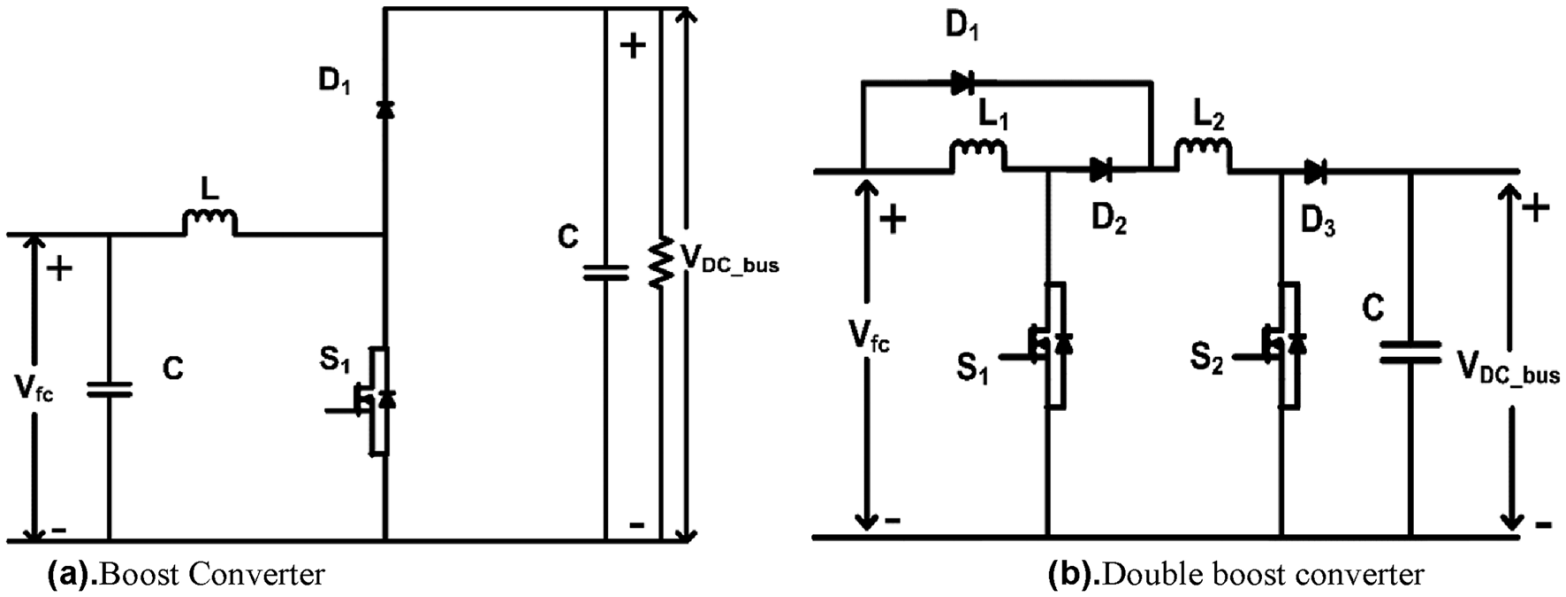
(**a**) Boost converter. (**b**) Double boost converter.

**Figure 4:**
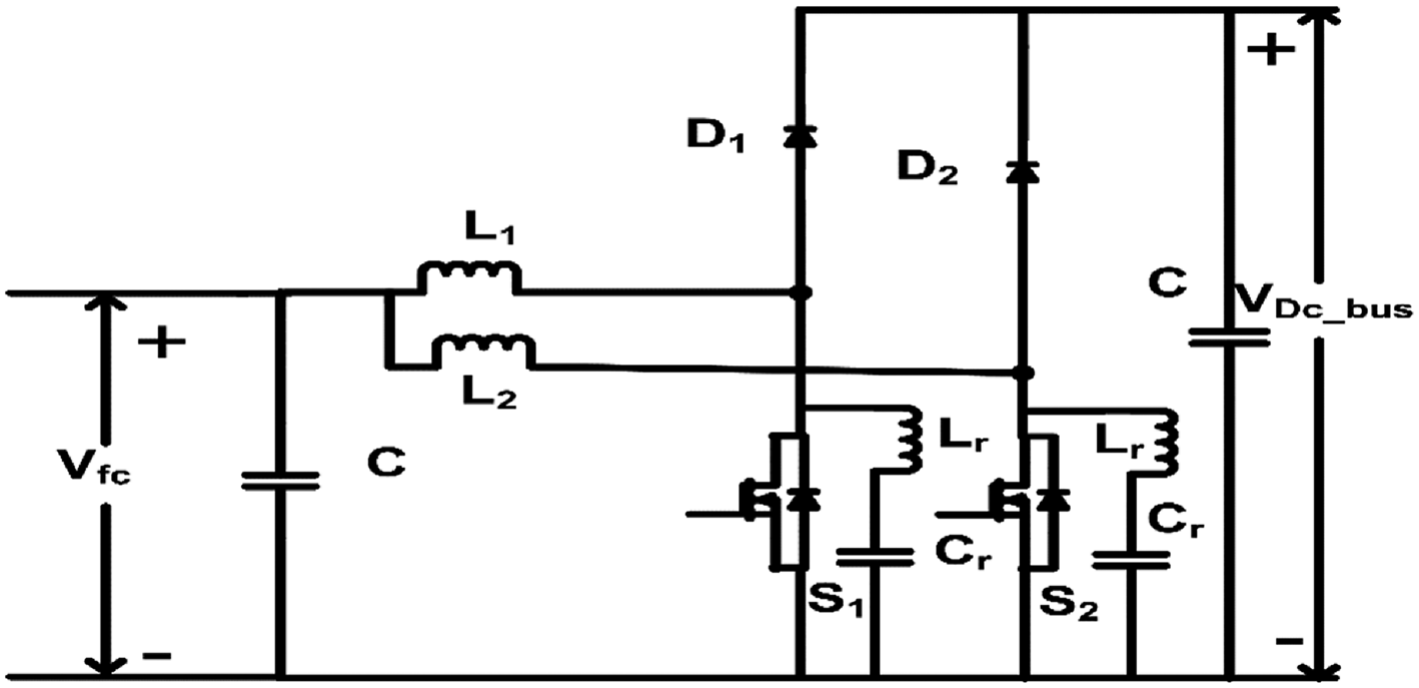
Proposed dual phase converter.

In this paper, a dual-phase converter is proposed as shown in Fig. 4 as an alternative, requiring fewer diodes and experiencing reduced switch stress compared to existing non-isolated DC to DC converters outlined in literature. To substantiate the superiority of this converter choice, two additional converters were identified^19,25^ as shown in Figs. 3a,b and 4 all three were subjected to simulation using common specifications. The results of these simulations are presented in the Table 2.

The no of components is less in the conventional boost however the voltage and current stress on the switches are high as shown in Table 1. The proposed converter has lesser device counts.

**Table 1:** Components comparison of non-isolated DC-DC topologies with the proposed topology.

Components	Boost converter^19^	Double boost converter^25^	Dual phase converter (proposed)
No. of inductor	1	2	2
No. of capacitor	1	1	1
No. of switches	1	2	2
No. of diodes	1	3	2

As shown in Table 2, switch voltage and current stress for the proposed converter is less compared to other two converters. The device count is also less compared to the double boost converter. Also, Zero voltage switching (ZVS) is proposed with the dual phase converter to improve the efficiency. Hence resonant dual phase converter is chosen for this application and its steady state analysis is discussed in the next section.

**Table 2:** Comparison of different non-isolated DC–DC topologies with the proposed topology.

Device stress in converter	Boost converter^19^ (6 to 24 V)	Double boost single phase converter^25^ (6 to 24 V)	Dual phase converter (6 to 24 V)
Duty cycle	0.75	0.6	0.75
Switch peak voltage	24 V	30 V	24 V
Diode peak voltage	24 V	9 V	24 V
Switch peak current	41.9 A	26.04 A	9.4 A
Switch RMS current	36.6 A	19.95 A	7.5 A
Diodes peak current	41.9 A	D_1_ = 26 A D_2_ = D_3_ = 26 A	9.4 A
Diodes RMS current	19.9A	D_1_ = 19.95 A D_2_ = D_3_ = 16.73 A	4.81 A
Inductor value	200 µH	86 µH	100 µH

## Steady state analysis and mode of operation

**Figure 5:**
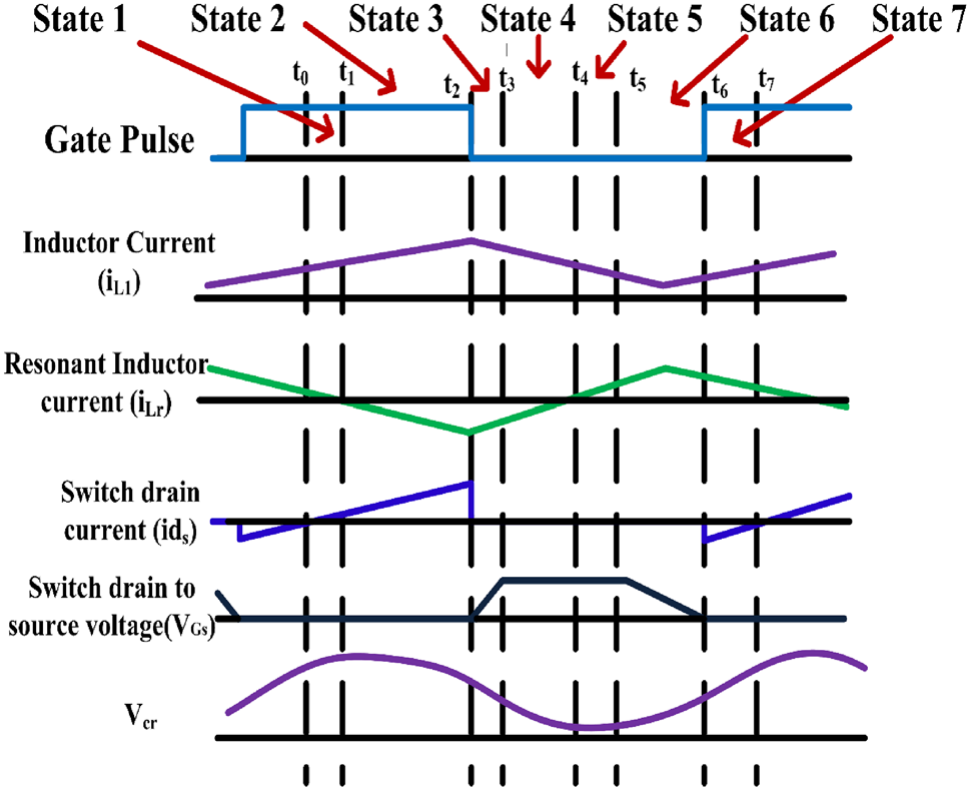
Theoretical steady state waveforms.

**Figure 6:**
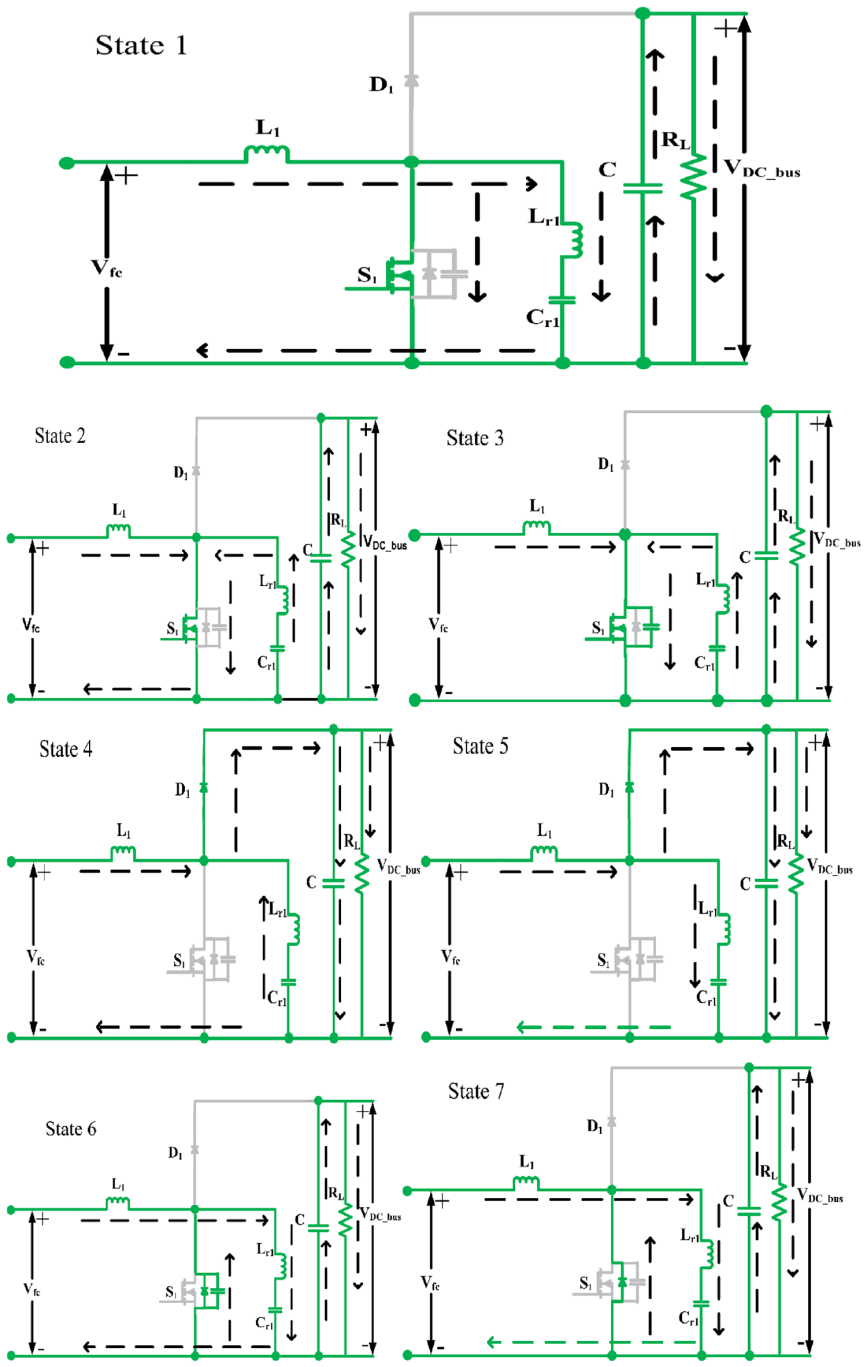
Modes of operation.

The efficiency of DC–DC converters diminishes significantly with hard switching, prompting the pursuit of alternatives to enhance performance. One such method is zero voltage switching (ZVS), which is integral to the proposed converter design. A resonant capacitor (Cr) and inductor (Lr) are incorporated into the circuit to establish LC resonance, facilitating ZVS when the resonant inductor current flows through the anti-parallel diode of the switch. To simplify analysis, focus is placed on a single leg of one phase, with the understanding that the principles apply universally. The soft switching and steady-state characteristics of the proposed converter are elucidated. Figure 5 depicts a segment of the converter operating in boost mode, illustrating various operating modes and their corresponding waveforms. Specifically, as shown in Fig. 6 there are seven distinct operating modes for the proposed converter, each elucidated within a single switching cycle. These modes delineate the sequential states of the converter, from the activation of the switch to the discharge and recharge of the resonant capacitor, ultimately ensuring optimized performance and efficiency across all phases of operation.

### State 1 $({{\varvec{t}}}_{0}\le {\varvec{t}}\le {{\varvec{t}}}_{1})$

In this phase initiates with the conduction of switch S_1_. The input inductor current L_1_ storing the energy. At the time the output capacitor supplying to the load. The current IL_1_ in the inductor circulates through the resonant circuit and switch S_1_. As the resonant capacitor achieves full charge, the current passing through the resonant inductor diminishes to zero. This signifies the completion of this stage, marked by the cessation of current flow through the resonant inductor.

1 $${i}_{Lr1}\left(t\right)={i}_{L1}\left({t}_{0}\right).cos{\omega }_{r}t- \frac{{V}_{Cr1}\left({t}_{0}\right)}{{Z}_{r}}.sin{\omega }_{r}t$$

2 $${V}_{Cr1}\left(t\right)= {V}_{Cr1}\left({t}_{0}\right)cos{\omega }_{r}t+{i}_{L1}({t}_{0}){Z}_{r}sin{\omega }_{r}t$$

Resonant angular frequency and characteristic impedance are provided as

$${\omega }_{r}=\frac{1}{\sqrt{{L}_{r1}{C}_{r1}}}{Z}_{r}=\sqrt{\frac{{L}_{r1}}{{C}_{r1}}}$$

### State 2 $({{\varvec{t}}}_{1}\le {\varvec{t}}\le {{\varvec{t}}}_{2})$

At time t_1_, the direction of the resonant inductor current reverses and combines with the main inductor current to pass through switch

3 $${i}_{Lr1}\left(t\right)=- \frac{{V}_{Cr1}({t}_{1})}{{Z}_{r}}sin{\omega }_{r}t$$

4 $${V}_{Cr1}\left(t\right)= {V}_{Cr1}\left({t}_{1}\right)cos{\omega }_{r}t$$

### State 3 $({{\varvec{t}}}_{2}\le {\varvec{t}}\le {{\varvec{t}}}_{3})$

When the switch S_1_ is in off state, this phase becomes operational. The voltage across the switch increases to match the output voltage V_DC_bus_.

5 $${i}_{Lr1}\left(t\right)=\frac{{C}_{1}}{{C}_{s2}}{i}_{L1}\left({t}_{2}\right)+\left({i}_{Lr1}\left({t}_{2}\right)-\frac{{C}_{1}}{{C}_{s2}}{i}_{L1}({t}_{2})\right)cos\omega t-\frac{{V}_{cr1}({t}_{2})}{Z}sin\omega t$$

6 $${V}_{Cr1}\left(t\right)=\frac{{i}_{L1}({t}_{2})}{{C}_{r1}+{C}_{s2}}t+\frac{{C}_{1}}{{C}_{s2}}{V}_{Cr1}\left({t}_{2}\right)\left[1+\frac{{C}_{s2}}{{C}_{r1}}cos\omega t\right]+\frac{{C}_{1}}{{C}_{r1}}Z\left({i}_{Lr1}\left({t}_{2}\right)-\frac{{C}_{1}}{{C}_{s2}}{i}_{L1}({t}_{2})\right)sin\omega t$$

7 $${V}_{Cs2}\left(t\right)=\frac{{i}_{L1}({t}_{2})}{{C}_{r1}+{C}_{s2}}t+\frac{{C}_{1}}{{C}_{s2}}{V}_{Cr1}\left({t}_{2}\right)\left[1-cos\omega t\right]+\frac{{C}_{1}}{{C}_{s2}}Z\left(\frac{{C}_{1}}{{C}_{s2}}{i}_{L1}\left({t}_{2}\right)-{i}_{Lr1}({t}_{2})\right)sin\omega t$$

where

$$\omega =\frac{1}{\sqrt{{L}_{r1}C}}Z=\sqrt{\frac{{L}_{r1}}{{C}_{1}}}{C}_{1}=\frac{{C}_{r1}{C}_{s2}}{{C}_{r1}+{C}_{s2}}$$

### State 4 $({{\varvec{t}}}_{3}\le {\varvec{t}}\le {{\varvec{t}}}_{4})$

At the time reaches t_3_, the voltage across the switch equals V_DC_bus_ The accumulated energy within the main inductor L_1_ and the resonant circuit triggers the activation of switch D_1_. This phase persists until the energy stored in the resonant capacitor Cr_1_ completely dissipates, altering the path of the resonant inductor current, iLr_1_.

8 $${i}_{Lr1}\left(t\right)={i}_{Lr1}\left({t}_{3}\right)cos{\omega }_{r}t+\frac{{V}_{high}-{V}_{cr1}({t}_{3})}{{Z}_{r}}sin{\omega }_{r}t$$

9 $${V}_{Cr1}\left(t\right)={V}_{high}-\left({V}_{high}-{V}_{Cr1}\left({t}_{3}\right)\right)cos{\omega }_{r}t+{i}_{Lr1}({t}_{3}){Z}_{r}sin{\omega }_{r}t$$

### State 5 $({{\varvec{t}}}_{4}\le {\varvec{t}}\le {{\varvec{t}}}_{5})$

The energy stored in the main inductor is channelled towards the load and the resonant circuit. Consequently, there is an elevation in the current within the resonant inductor as its direction undergoes a change. Simultaneously, the current flowing through the main inductor, IL_1_, diminishes.

10 $${i}_{Lr1}\left(t\right)=\frac{{V}_{high}-{V}_{cr1}({t}_{4})}{{Z}_{r}}sin{\omega }_{r}t$$

11 $${V}_{Cr1}\left(t\right)={V}_{high}-\left({V}_{high}-{V}_{Cr1}\left({t}_{4}\right)\right)cos{\omega }_{r}t$$

### State 6 $({{\varvec{t}}}_{5}\le {\varvec{t}}\le {{\varvec{t}}}_{6})$

This phase operation commences as the stored energy in the parasitic capacitor discharges through the LC resonant circuit. Consequently, the voltage across the switch descends below V_DC_bus_, leading to the deactivation of switch D_1_. This marks the conclusion of state 6.

12 $${i}_{Lr1}\left(t\right)=\frac{{V}_{high}-{V}_{cr1}({t}_{4})}{{Z}_{r}}sin{\omega }_{r}t$$

13 $${V}_{Cr1}\left(t\right)={V}_{high}-\left({V}_{high}-{V}_{Cr1}\left({t}_{4}\right)\right)cos{\omega }_{r}t$$

14 $${i}_{Lr1}\left(t\right)=\frac{{C}_{1}}{{C}_{s2}}{i}_{L1}\left({t}_{5}\right)\left(1+\frac{{C}_{s2}}{{C}_{r1}}cos\omega t\right)+\frac{{V}_{high}-{V}_{cr1}({t}_{5})}{Z}sin\omega t$$

15 $${V}_{Cr1}\left(t\right)=\frac{{i}_{L1}\left({t}_{5}\right)}{{C}_{r1}+{C}_{s2}}t+{V}_{Cr1}\left({t}_{5}\right)+\frac{{C}_{1}}{{C}_{r1}}\left({V}_{high}-{V}_{Cr1}\left({t}_{5}\right)\right)\left(1-cos\omega t\right)+{\left(\frac{{C}_{1}}{{C}_{r1}}\right)}^{2}{i}_{L1}\left({t}_{5}\right)Zsin\omega t$$

16 $${V}_{Cs2}\left(t\right)=\frac{{i}_{L1}\left({t}_{5}\right)}{{C}_{r1}+{C}_{s2}}t+\frac{{C}_{1}}{{C}_{s2}}{V}_{Cr1}\left({t}_{5}\right)\left[1-cos\omega t\right]+\frac{{C}_{1}}{{C}_{r1}}{V}_{high}\left(1+\frac{{C}_{r1}}{{C}_{s2}}cos\omega t\right)-\frac{{C}_{1}}{{C}_{r1}+{C}_{s2}}{i}_{L1}\left({t}_{5}\right)Zsin\omega t$$

### State 7 $({{\varvec{t}}}_{6}\le {\varvec{t}}\le {{\varvec{t}}}_{7})$

This state commences as the current iLr_1_ from the resonant inductor traverses through the antiparallel diode of switch S_1_. This action ensures the fulfilment of zero-voltage switching for switch S_1_.

17 $${i}_{Lr1}\left(t\right)={i}_{Lr1}\left({t}_{6}\right)cos{\omega }_{r}t- \frac{{V}_{Cr1}({t}_{6})}{{Z}_{r}}sin{\omega }_{r}t$$

18 $${V}_{Cr1}\left(t\right)= {V}_{Cr1}\left({t}_{6}\right)cos{\omega }_{r}t+{i}_{Lr1}({t}_{6}){Z}_{r}sin{\omega }_{r}t$$

## Design of passive elements

### Inductor design (L_1_, L_2_)

The purpose of the inductors L_1_ and L_2_ are to reduce the input current ripple. Considering input current ripple ($\Delta IL)$ of 1%.

19 $$Main inductor value=\frac{{V}_{Fc}*D}{2*\Delta IL*{f}_{switch}}$$

By using equation (19)inductors values are computed and it is 100mH

### Capacitor design

Capacitor is connected at the output side of converter to reduce the output voltage ripple. When the proposed converter operates in boost mode, capacitor design given by considering output voltage ripple of 1%

20 $$C=\frac{D}{{R}_{L}*\frac{\Delta {V}_{load}}{{V}_{load}}*{f}_{switch}}$$

By using equation (20)Capacitor values are computed and it is 650 µF

### Resonant inductor design (L_r_)

When peak value of resonant inductor current (i_Lrpeak_) greater than or equal to minimum value of main inductor current (i_Lmin_), resonant condition is satisfied. Equation represents resonant condition, from this value of resonant inductor is designed as follows.

21 $${I}_{Lmin}\le {I}_{Lr peak}$$

22 $${I}_{Lmin}=\left(\frac{{V}_{load}}{{V}_{FC}}\right)*Io-\frac{{V}_{load}*{T}_{on}}{2*L}$$

23 $${I}_{Lr peak}=\frac{{V}_{load}*{T}_{on}}{2{L}_{r}}$$

24 $${L}_{r}\le \frac{{V}_{load}*{T}_{on}*L}{(2Gv*{V}_{FC}*L-*{T}_{on})}$$

By using equation (24)resonant inductor values are computed and it is 6µH

Where L is the input inductor, voltage gain $Gv$ and Switch ON ${T}_{on}$

### Resonant capacitor design (C_r_)

For achieving ZVS operation of the converter, the resonant frequency must be lower than or equal to half of the switching frequency. In this study, the resonant capacitor value is determined by setting the resonant frequency to 0.4 times the switching frequency, as specified in the equation

25 $$\frac{2.5}{2\pi \sqrt{{C}_{r{*L}_{r}}}}\le {f}_{switch}$$

26 $${C}_{r}\le \frac{6.25}{4*{L}_{r}*{\pi }^{2}{{f}_{switch}}^{2}}$$

By substituting resonant inductor value from equation (26), the value of resonant capacitor is designed and computed it is 2.24 µF

## Simulation results

**Figure 7:**
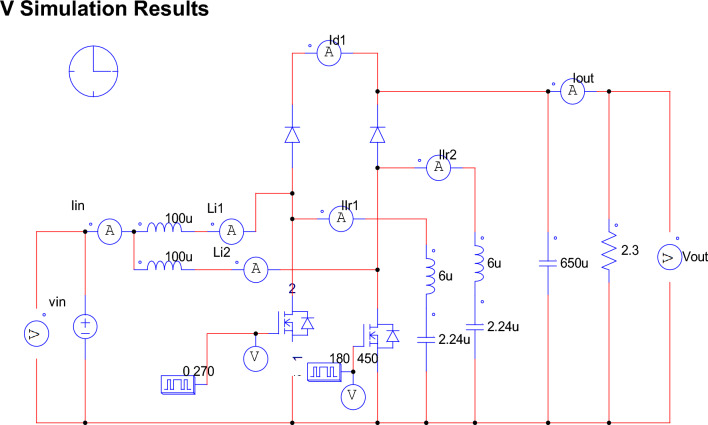
Simulation circuit for dual phase converter.

**Figure 8:**
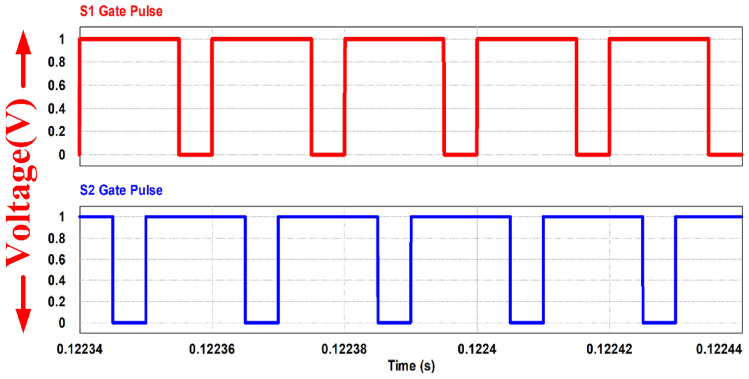
Gate pulses to the switch.

**Figure 9:**
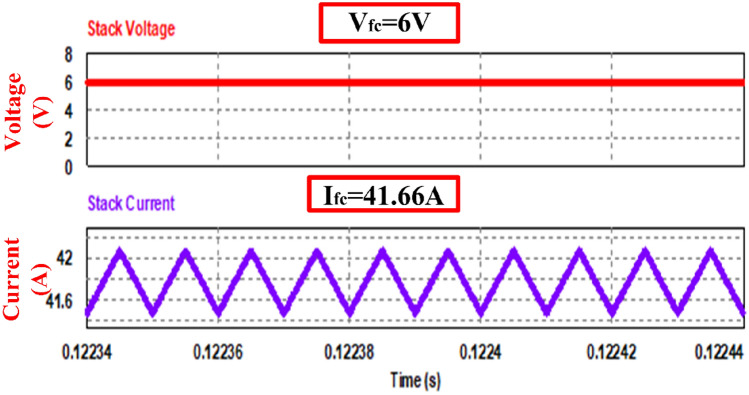
Stack voltage and current waveform.

**Figure 10:**
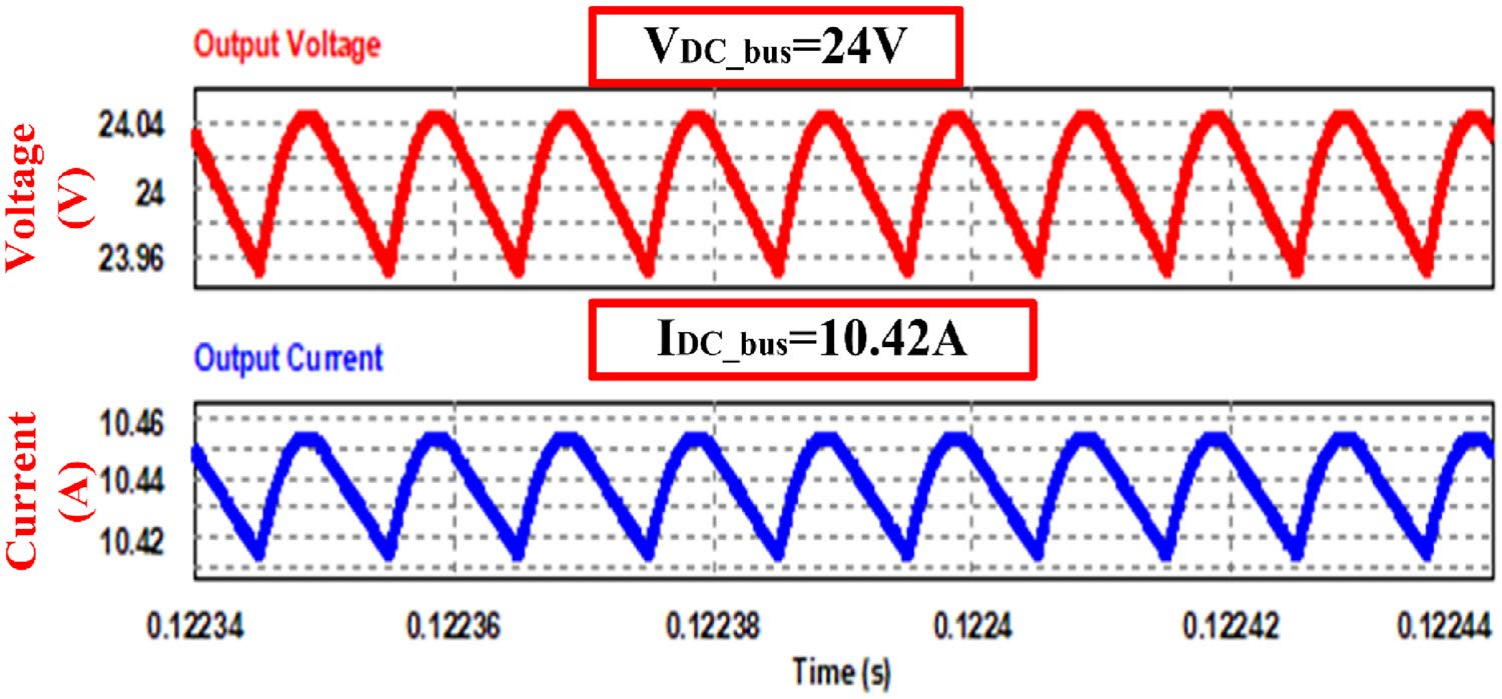
Output voltage and output current waveform.

**Figure 11:**
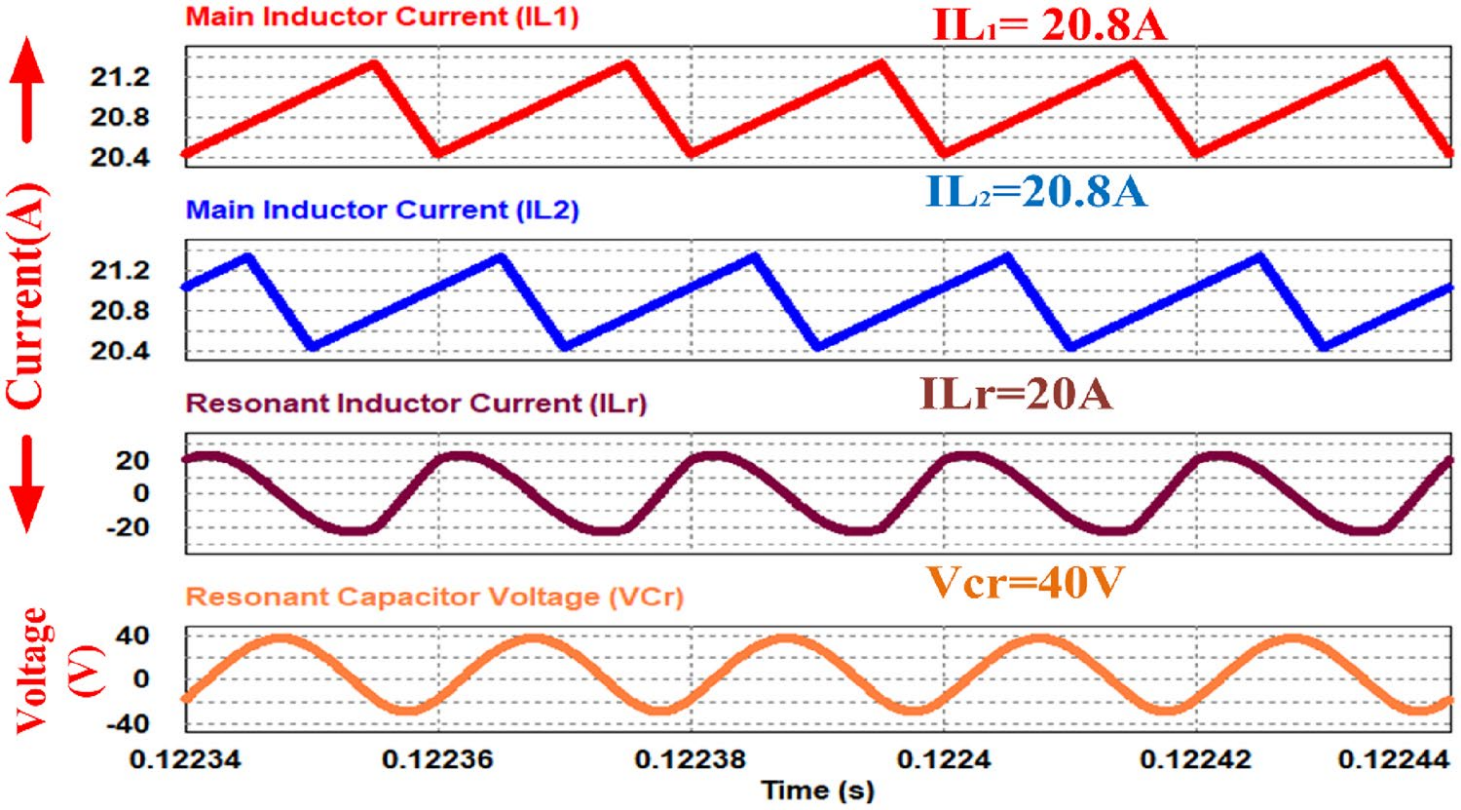
Waveforms of passive elements.

**Figure 12:**
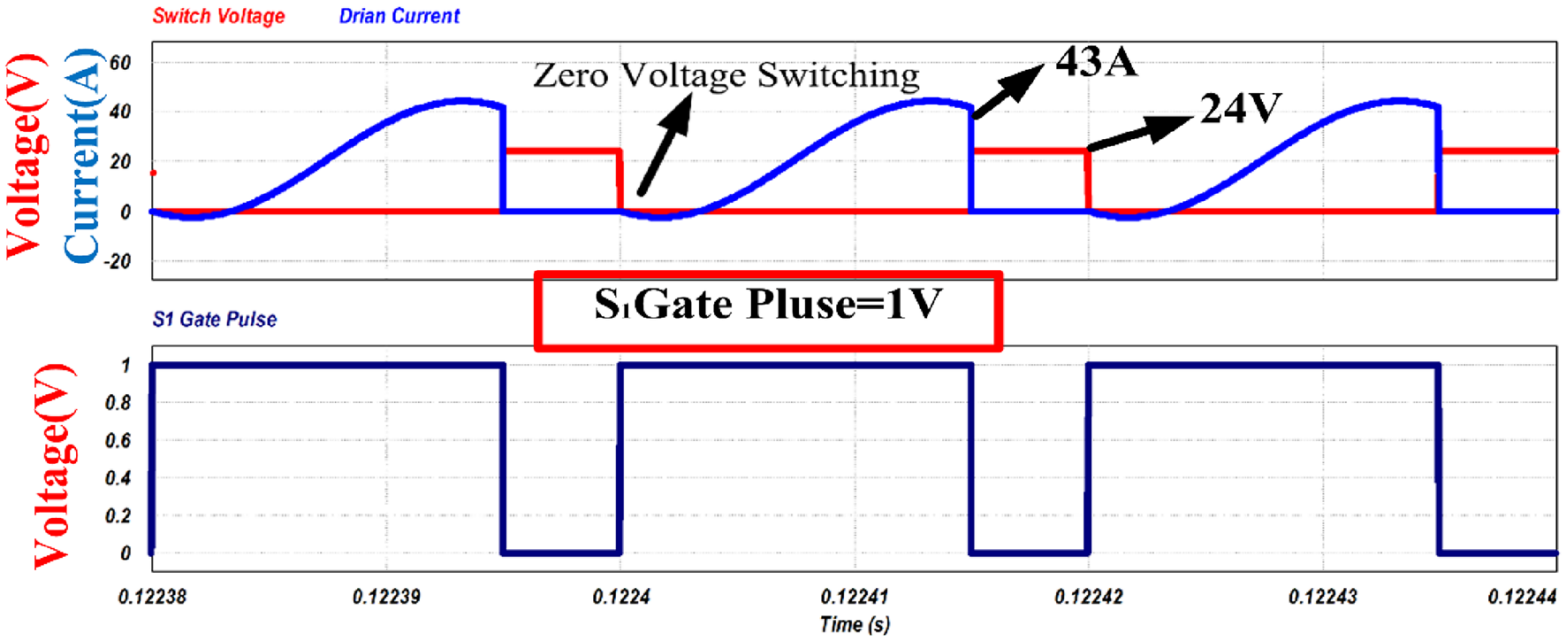
ZVS condition of switch S_1_.

**Figure 13:**
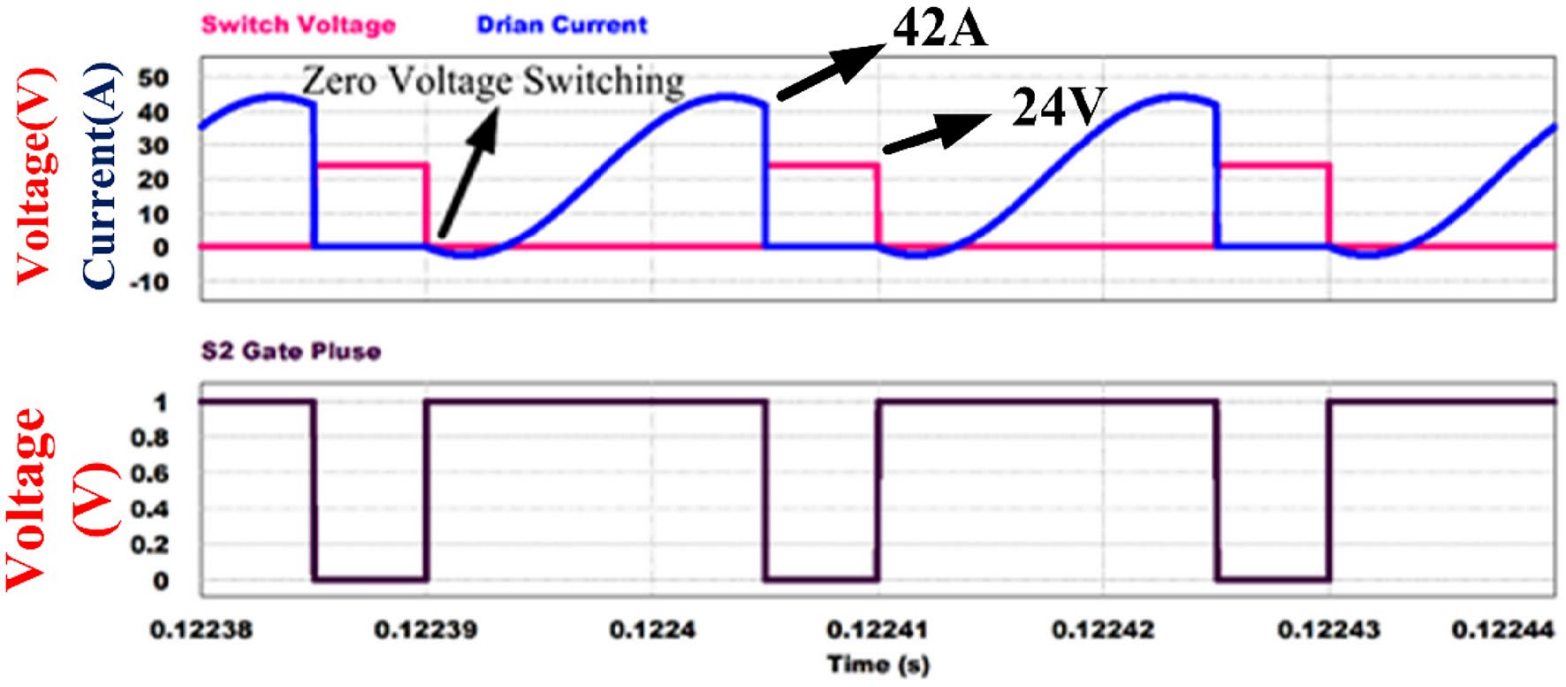
ZVS condition of switch S_2_.

In Section 5, the theatrical waveform as shown in Fig. 5 presented serves to demonstrate the performance of the proposed converter. Subsequently, the proposed converter is simulated using PSIM software 9.1.1.400, and the obtained results are thoroughly analyzed. Figure 7 displays the simulated circuit of the proposed converter along with its corresponding simulation settings. The specifications are detailed in Table 3. Simulation outcomes for two scenarios V_fc_ = 6 V at full load (250 W) and V_fc_ = 7 V at half load (125 W) are depicted in Figs. 8, 9, 10, 11, 12 and 13. These simulation results are in alignment with the theoretical waveforms, validating the steady-state analysis of the proposed converter as described in Section 5.

**Table 3:** Simulation parameters.

Parameter	Values
Input voltage	6 V
Output voltage	24 V
Duty ratio	0.75 and 0.25
Load resistance	2.304 ohms
Power rating	250 W
Switching frequency	50 kHz
Resonant inductor	6 µH
Resonant capacitor	2.24 µF
Main inductor (L1 and L2)	100 µH
Boost capacitor (C)	650µF

Figure 8 shows the gate signals of the switches with switching frequency of 50 kHz and duty cycle of 0.75. Figure 9 shows stack voltage and current for an input power of 250 W. (V_fc_ = 6 V & I_fc_ = 41.66A) the input ripple is assumed 1%, and it evident that the stack current ripple is within the limit.

Figure 10 shows simulation results for V_fc_—6 V delivering peak power of 250 W to the load. For an input stack voltage of 6 V, an (V_DC_bus_) output of 24 V obtained and the ripple is 0.09 V which is within the limit. The output current (I_DC_bus_) is 10.42A.

Figure 11 displays the current waveforms across two input inductors, as well as the resonant inductor, and the voltage across the resonant capacitor. The currents, i_S1_ and i_S2_, passing through switches S_1_ and S_2_, respectively, correspond precisely to the steady-state waveforms depicted in the figure. Furthermore, the currents through the boost inductors, IL_1_ and IL_2_, exhibit a phase shift of 180 degrees, resulting in a reduced ripple magnitude in the input or source current.

Figures 12 and 13 shows the switch gate pulses, switch drain to source voltages and switch drain currents. The anti-parallel body diode of the switches S_1_ and S_2_ conducts before it starts conducting current through it. It results in ZVS on of the switches. It verifies the design of the converter explained in Section V.

**Figure 14:**
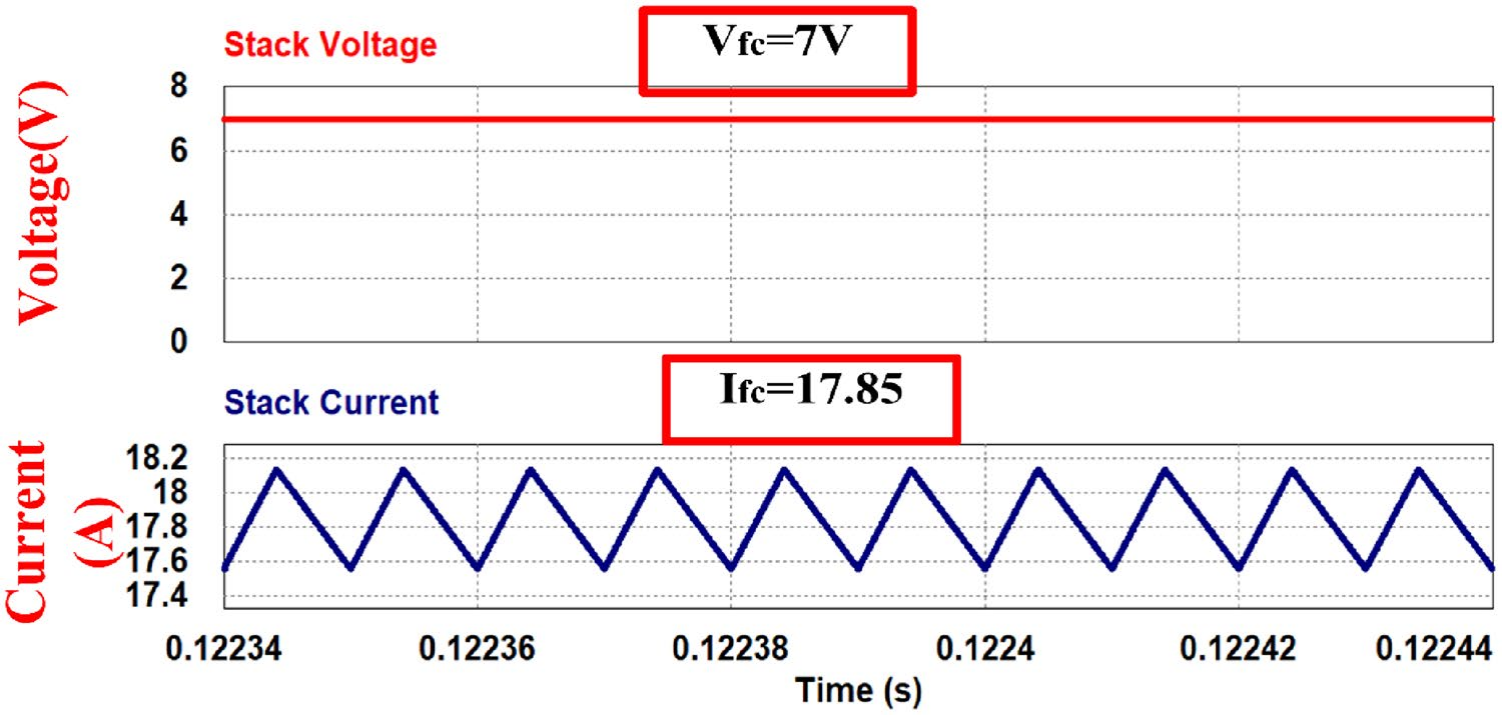
Stack voltage and current waveform.

**Figure 15:**
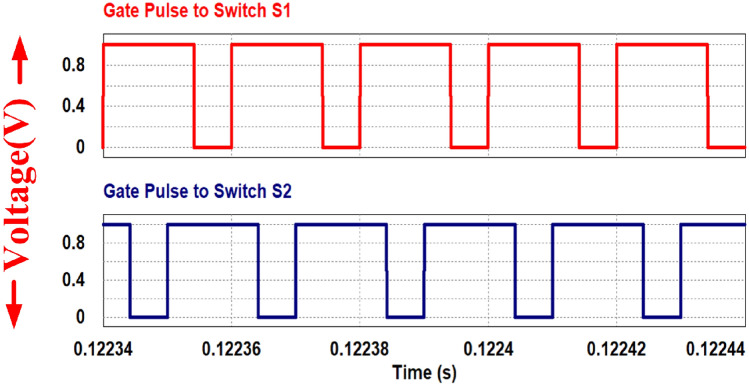
Gate pulses to the switch.

**Figure 16:**
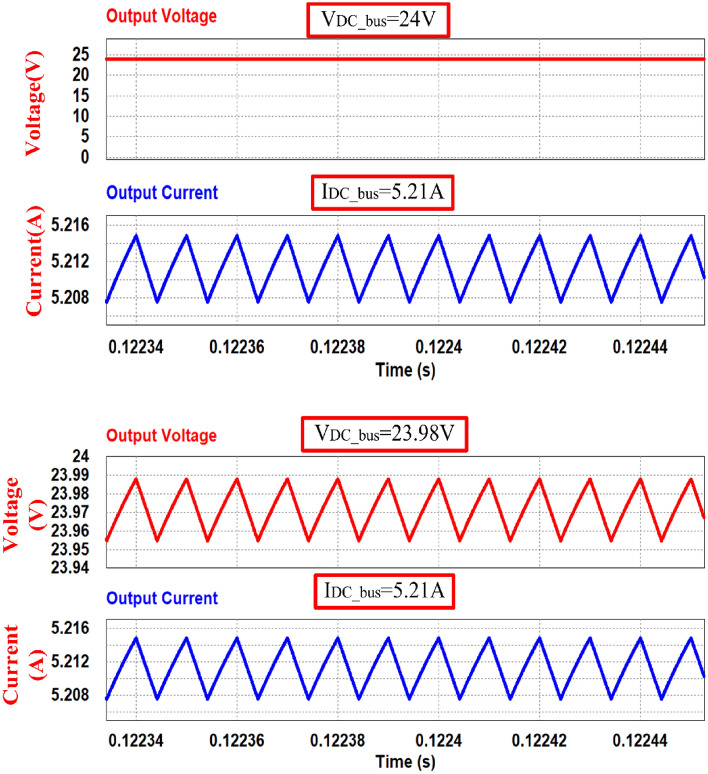
Load voltage and current waveform.

**Figure 17:**
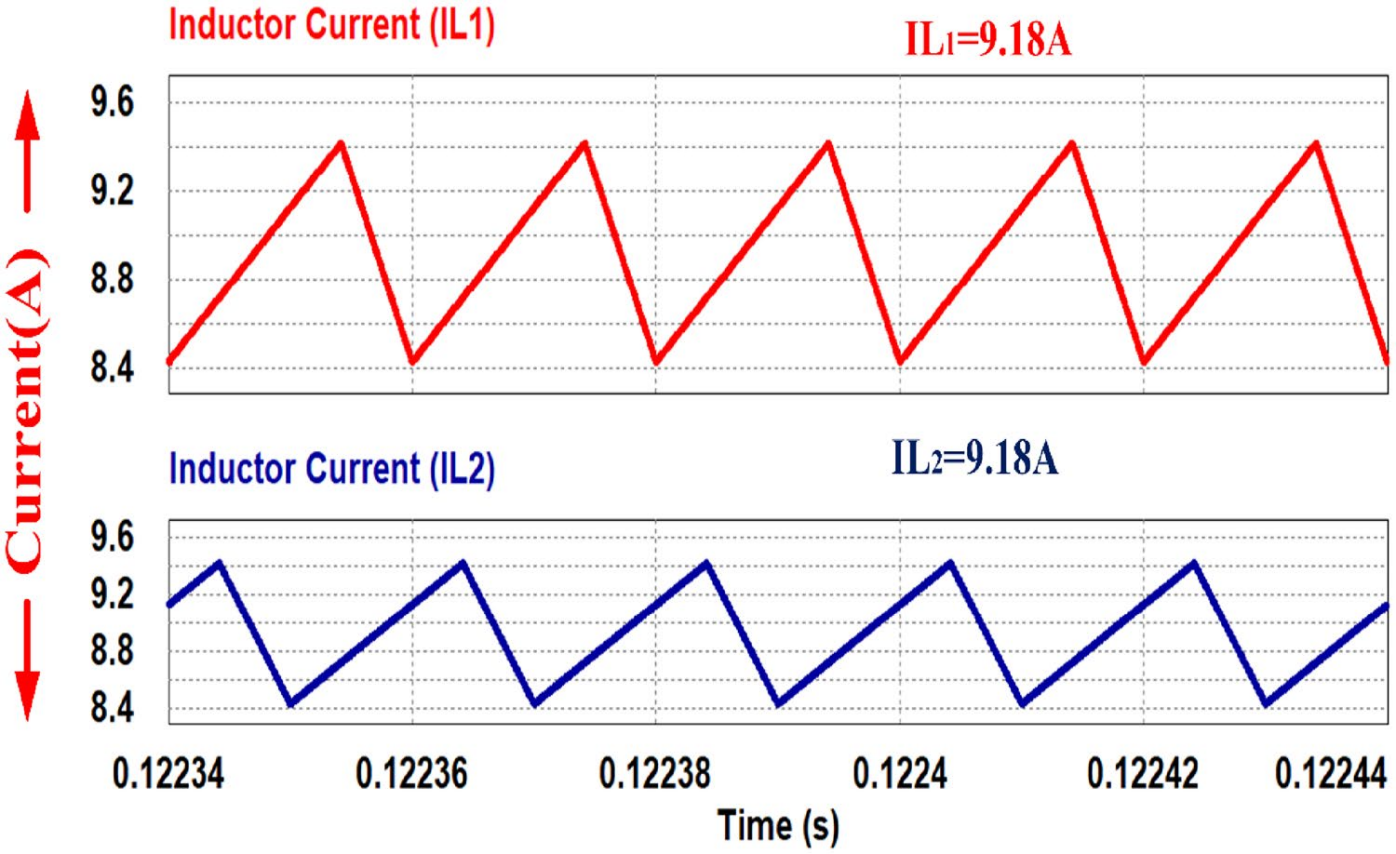
Inductor current waveforms (IL_1_&IL_2_).

As the load decreases, there's a tendency for the fuel cell stack voltage to increase. To assess the performance of the proposed converter under these conditions, the converter is simulated with half the load. The load resistance is doubled, and it's assumed that the stack voltage increases from 6 V at full load to 7 V as shown in Fig. 2. With the resistance doubled, the current is halved, indicating a half-load condition. Simulation waveforms are presented for V_fC_ = 7 V and 50% power (half load) in Figs. 14, 15, 16 and 17.

Simulation is carried out assuming stack voltage is increased to 7 V while removing the 50% of the load. Figure 14 shows the stack voltage and current waveforms. The stack voltage is 7 V and stack current is 17.85A. Figure 15 shows gate signals to the switches. The duty cycle reduced to 70.8%. To main the same output voltage.

The output voltage (V_DC_bus_) and output current (I_DC_bus_) waveforms are shown in Fig. 16 gives the simulation results for 50% power when 4.608-Ω load is connected to the converter. Converter maintains same output voltage of 24 V obtained and the ripple is 0.03 V which is within the limit. And output current reduced to 5.21 A which represents 50% of output power (125 W).

Figure 17 shows the inductor current waveforms when the load power is 50%. IL_1_ and IL_2_ shares the current equally and has 180 degree phase shift which ensure the ripple cancellation at the stack input.

## Experimental results

**Figure 18:**
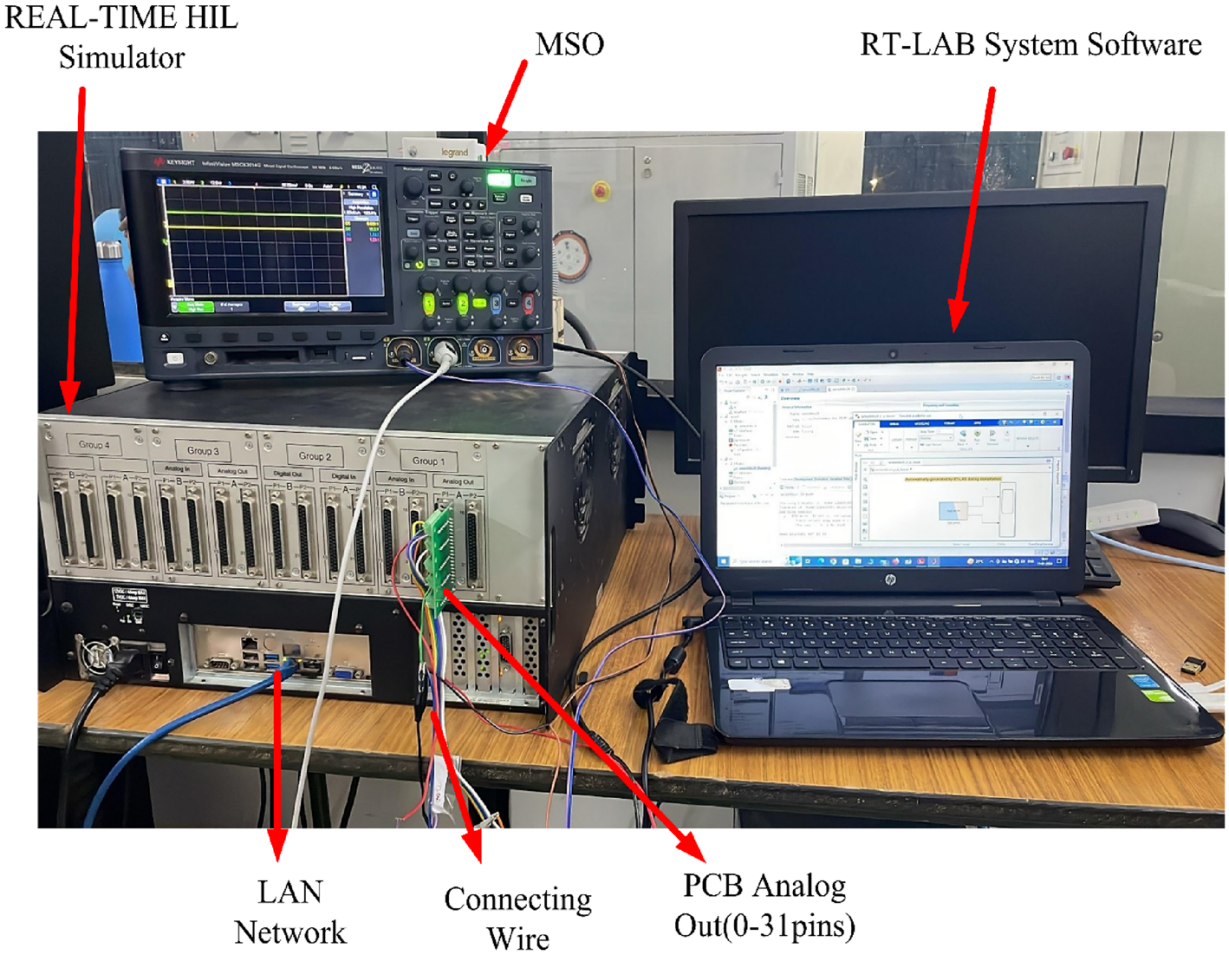
HIL experimental setup for the propoosed dual phase converter.

The validation of the proposed converter was conducted using the HIL simulator OP5700, RT-LAB, PCB-E06-0560, MSOx3014G, and other probes. The PCB facilitates communication between the controller and simulation by means of digital inputs and analog outputs. Figure 18 illustrates the arrangement of the real-time execution setup. The deployment of HIL systems is a common practice in engineering for conducting real-time simulations prior to the implementation of prototype testing. Stacks provide the ability to quickly generate and coordinate prototypes. In order to provide real-time clock speeds, the controller and plant are positioned within the OPAL-RT system. This procedure can be regarded as a real-time simulation because of its high-speed sampling rate, which ranges from nanoseconds to microseconds, using OPAL-RT. The user's personal computer is utilized to perform the simulator commands of the RT-LAB. The prototype is edited, built, loaded, and executed using RT-LAB. Table 4 presents the specifications of OP5700 Simulator HIL stack and Table 5 shows circuit parameters of the proposed converter.

**Table 4:** Specifications of the Hardware-in-loop (HIL) system using the OP5700 simulator.

Device name	OP5700 (HIL) simulator
Processing	200 ns–20 microsecond
Input/output LINES	Featuring 256 Input / Output lines, the system can be routed to eight analog or digital, 16, or 32 channels
Ports and communication	High-speed communication is facilitated through 16 SFP sockets supporting up to 5 GBps
Field programmable gate array	7 Field Programmable Gate Array ON VC707 board Xilinx® Virtex®
Input/output connectors	HIL system incorporates four panels of DB37 connectors
Laptop interface	Through LAN Port
Monitoring	Four panels of RJ45 connectors for monitoring
Ports & communication	High-speed communication is facilitated through 16 SFP sockets supporting up to 5 GBps
Power specification	Operates with a power input of 100–240 VAC, 50–60 Hz, drawn 10/5 A, resulting in a power output of 600W

**Table 5:** Circuit components.

Name of the component	Parameter
Main inductor L_1_ & L_2_	100 µH
Capacitor C	650 µF
Resonant inductor (Lr)	6 µH
Resonant capacitor (Cr)	2.24 µF
Operating frequency	50 kHz
Output power	250 W
Stack voltage	6 V
Output voltage	24 V

**Figure 19:**
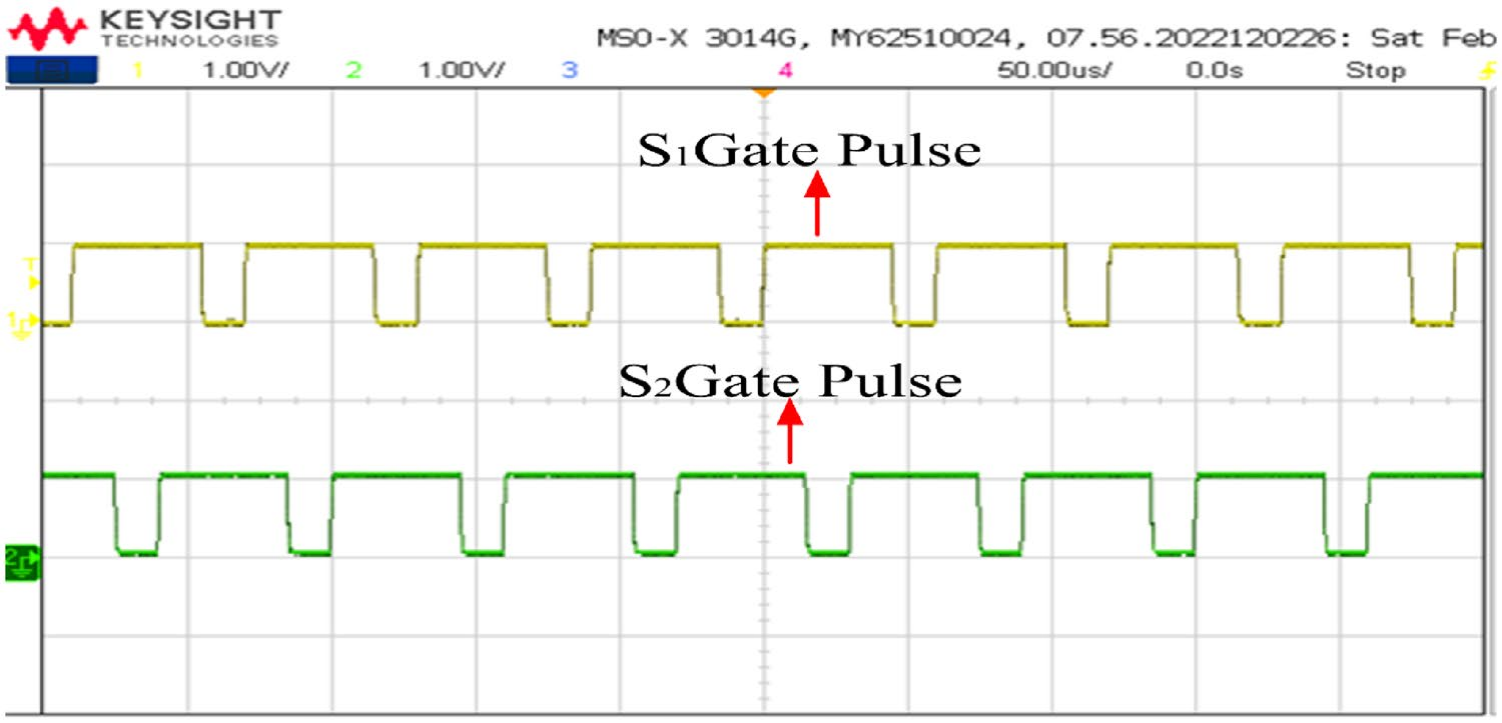
Switching gate pulse S_1_ and S_2_.

The PWM driving signals for the switches are depicted in Fig. 19. Channel 1 is the gate pulse for switch 1 and channel 2 is the gate pulse for switch 2. The Figure illustrates that the converter operates at a switching frequency of 50 kHz with 75% duty cycle. To ensure equal division of inductor currents and for ripple cancellation, a 180-degree phase shift is maintained between the switches.

**Figure 20:**
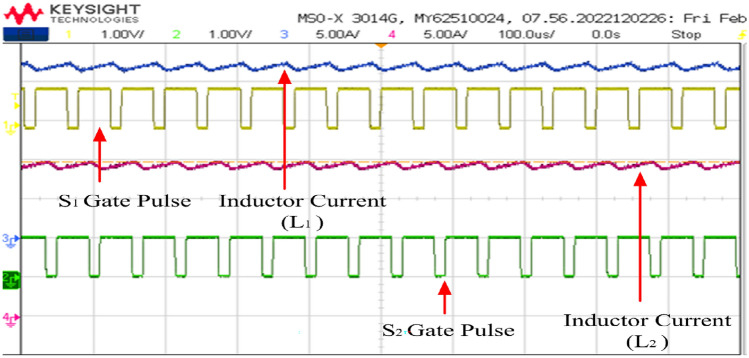
Inductor current waveforms with switching pulse.

The currents flowing through the input inductors, along with the gating signals for switches S_1_ and S_2_, are illustrated in Fig. 20. The converter is operating at continuous conduction (CCM) mode. The ripple in the current waveforms measures less than 0.203 A, as precisely calculated theoretically. It's evident that the two currents are out of phase by 180°, resulting in a ripple of twice the switching frequency in the input current. This ripple cancellation mechanism significantly reduces the magnitude of the input current ripple, thereby minimizing the size requirement for the input capacitor connected across the fuel cell stack. Lower ripple ensures a longer lifespan for the fuel cell stack.

**Figure 21:**
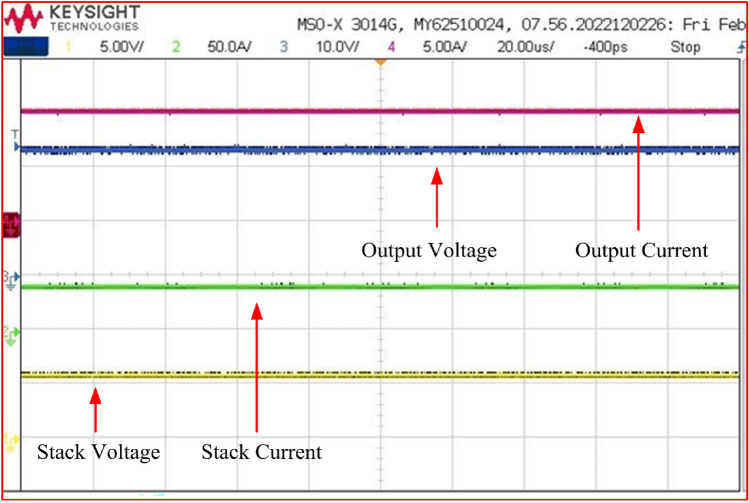
Input and output VI waveforms.

The data presented in Fig. 21 illustrates the voltage and current waveforms for both input and output. Channel 1 corresponds to the input stack voltage, which measures 6 V, while Channel 2 depicts the input stack current, averaging at 42 amperes. Channel 3 represents the DC grid bus voltage, and Channel 4 displays the DC grid current. Upon inspection of the Figure, it becomes evident that when the input stack voltage is maintained at 6 V, the output DC bus voltage increases to 24 V, accompanied by an output current of 10.3 amperes. Impressively, this configuration yields an efficiency rating of approximately 98.1%.

**Figure 22:**
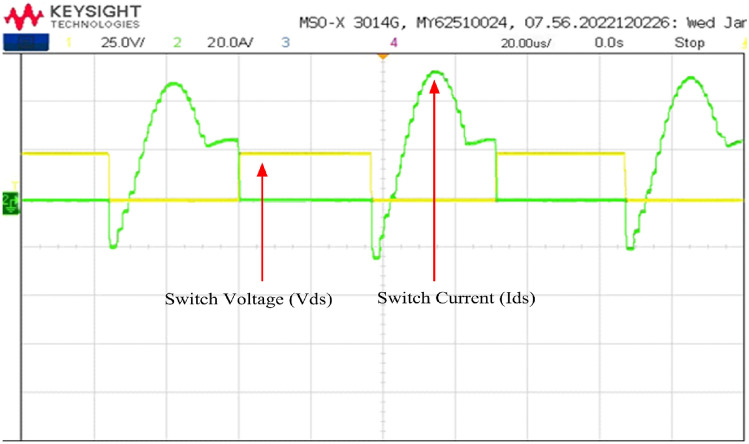
Zero voltage switching.

In Fig. 22, the drain-to-source voltage (V_DS_) and drain current for switch 1 are illustrated. The appearance of negative current suggests that the conduction of anti-parallel body diode across the switch, resulting in a voltage across the switch 1 is zero (V_DS_ = 0). Once the gate signal is applied, the device's voltage has already dropped to zero. Following this, the switch begins conducting, leading to a positive current in the waveform. The switch effectively takes over the current with continuous zero voltage across it, facilitating zero-voltage switching (ZVS) turn-on.

**Figure 23:**
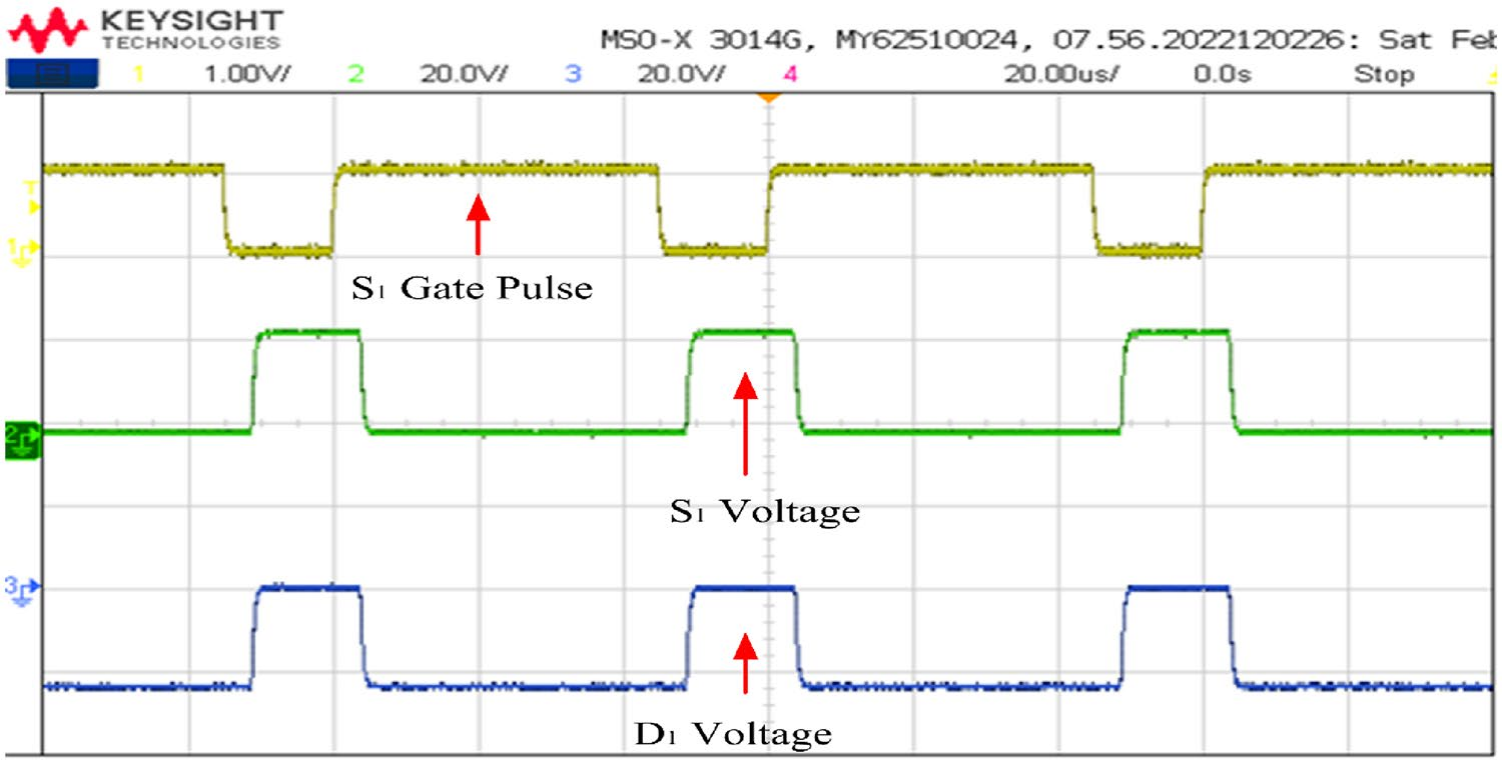
Switch and diode voltage waveforms.

**Figure 24:**
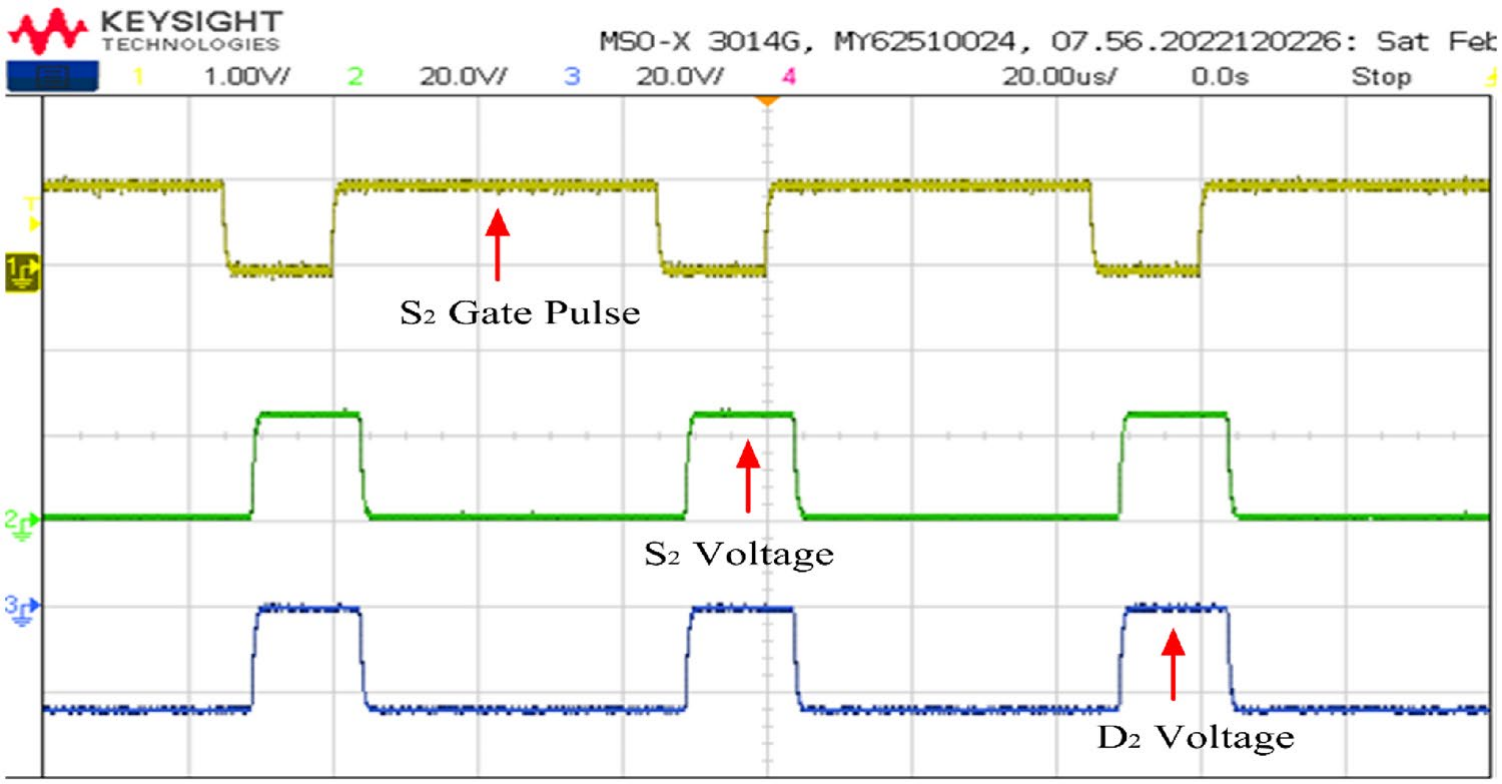
Switch and diode voltage waveforms.

Figures 23 and 24 depict voltage waveforms for both switches and diodes along with the gate pulses. Sequentially, the waveforms include the S_1_ gate pulse, drain-to-source voltage (V_DS__S_1_), and voltage across diode V_D1_. Analysis of Fig. 23 reveals that during the gate pulse activation, the voltage across switch 1 drops to zero, indicating its conduction state. Channel 3 confirms that diode D_1_ is in an off state, with the maximum voltage it blocks being -24 V. After the gate pulse is removed, switch 1 deactivates, allowing the diode D_1_ to begin conducting. The maximum voltage blocked by the switch is nearly 24 V. The same principles apply to Fig. 24.

**Figure 25:**
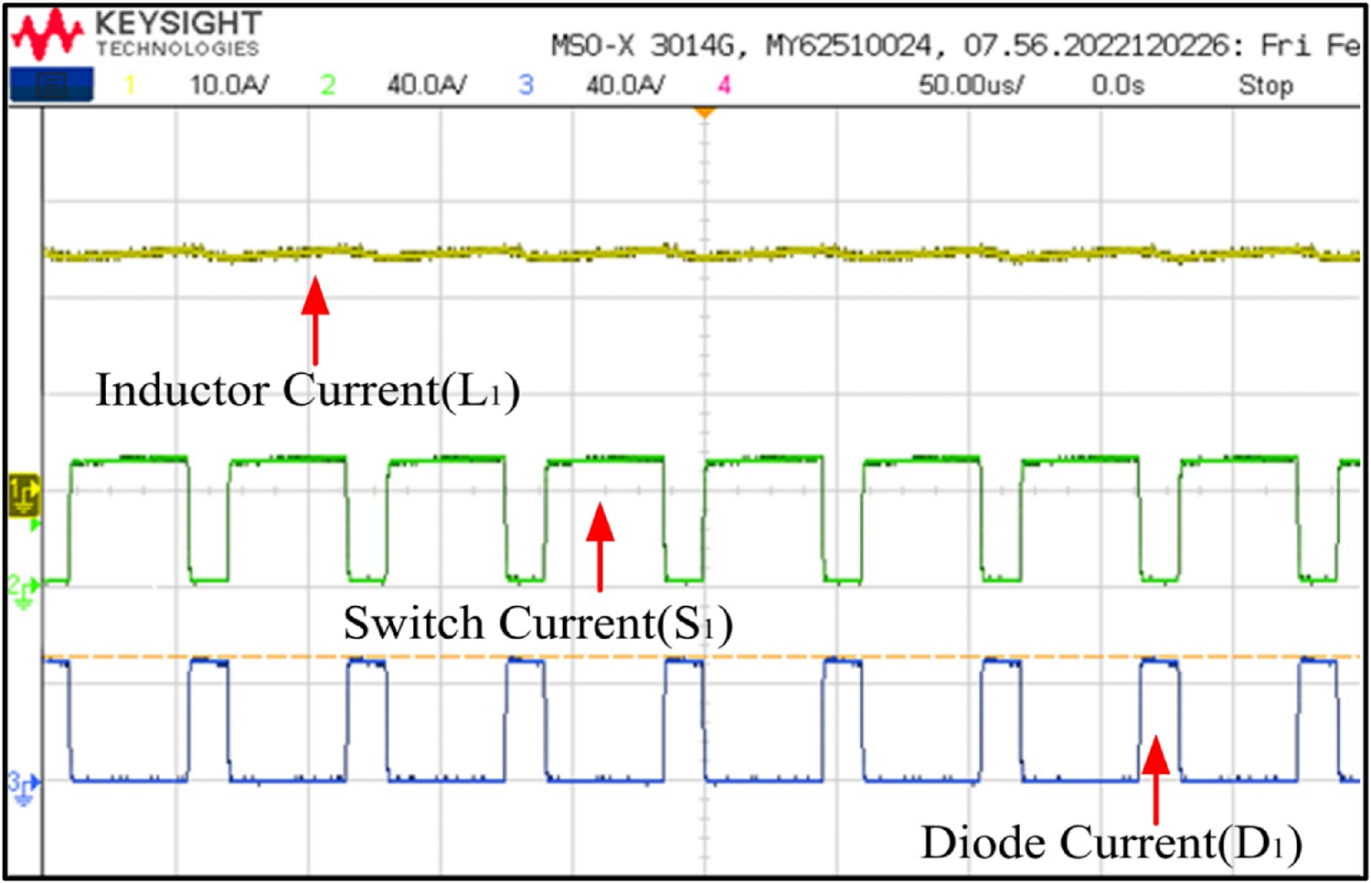
Switch and diode current waveforms.

**Figure 26:**
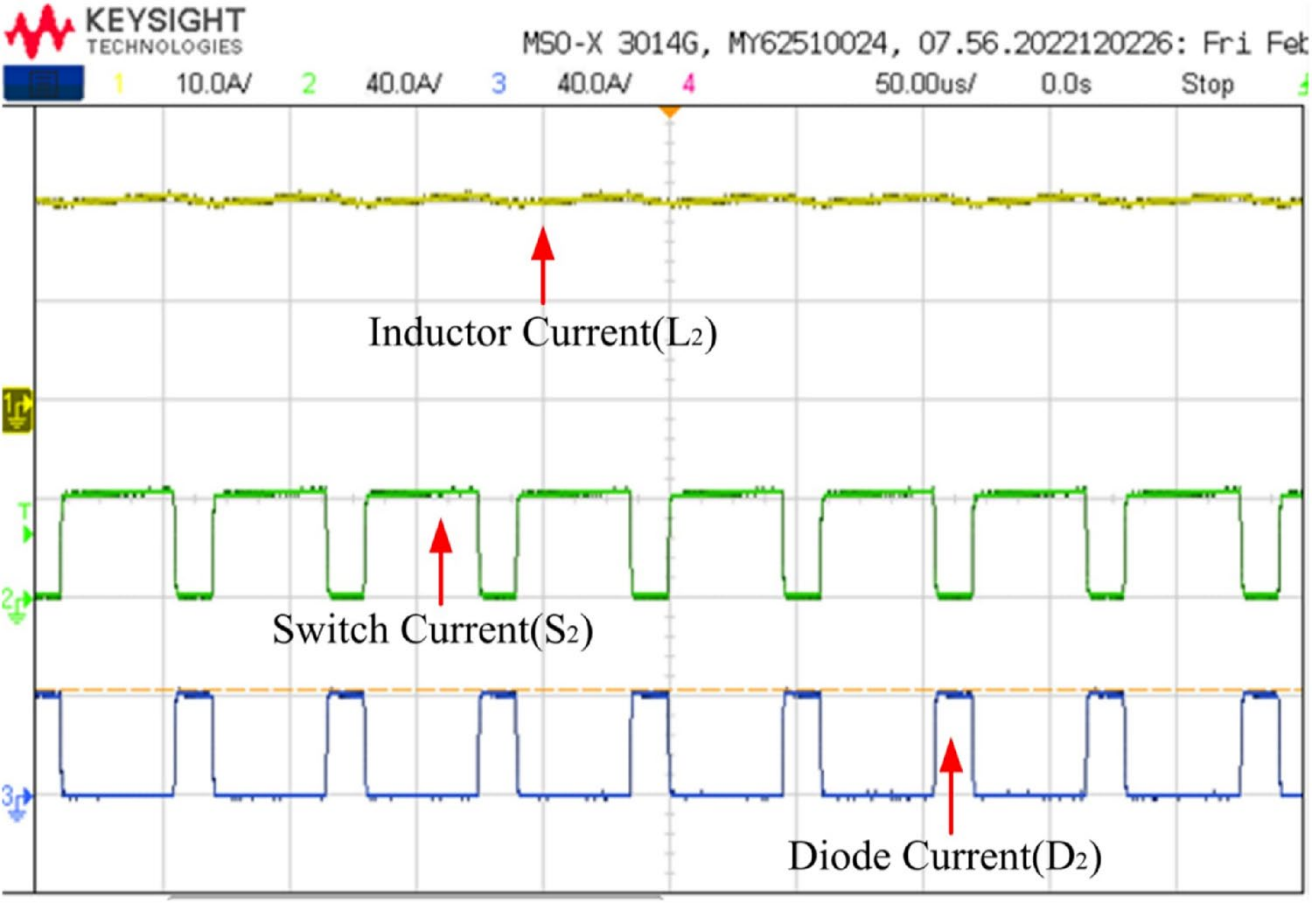
Switch and diode current waveforms.

Figures 25 and 26 depict switch and current waveforms. The waveforms include inductor currents, switch currents, and diode currents, arranged from top to bottom. In Fig. 25, Channel 1 illustrates the inductor current, IL_1_, fluctuating between I_min_ and Imax when S_1_ is switched on, and from I_max_ to I_min_ when S_1_ is turned off. Channel 2 and 3 represents the switch 1 and diode 1 current waveforms. Observing Fig. 25, it becomes apparent that switch 1 carries the same IL_1_ current. Upon activation of S_1_, the current begins at I_min_, reaches I_max_, and then S_1_ switches off, transferring the current from I_max_ to I_min_ through diode 1. At steady state the I _max_ and I_min_ values are remain same. The same explanation apply to Fig. 26.

The experimental waveforms align closely with the anticipated steady-state operating patterns as predicted analytically and simulated. Under full load conditions, the converter maintains ZVS (soft-switching), ensuring efficient operation.

### Half load condition

**Figure 27:**
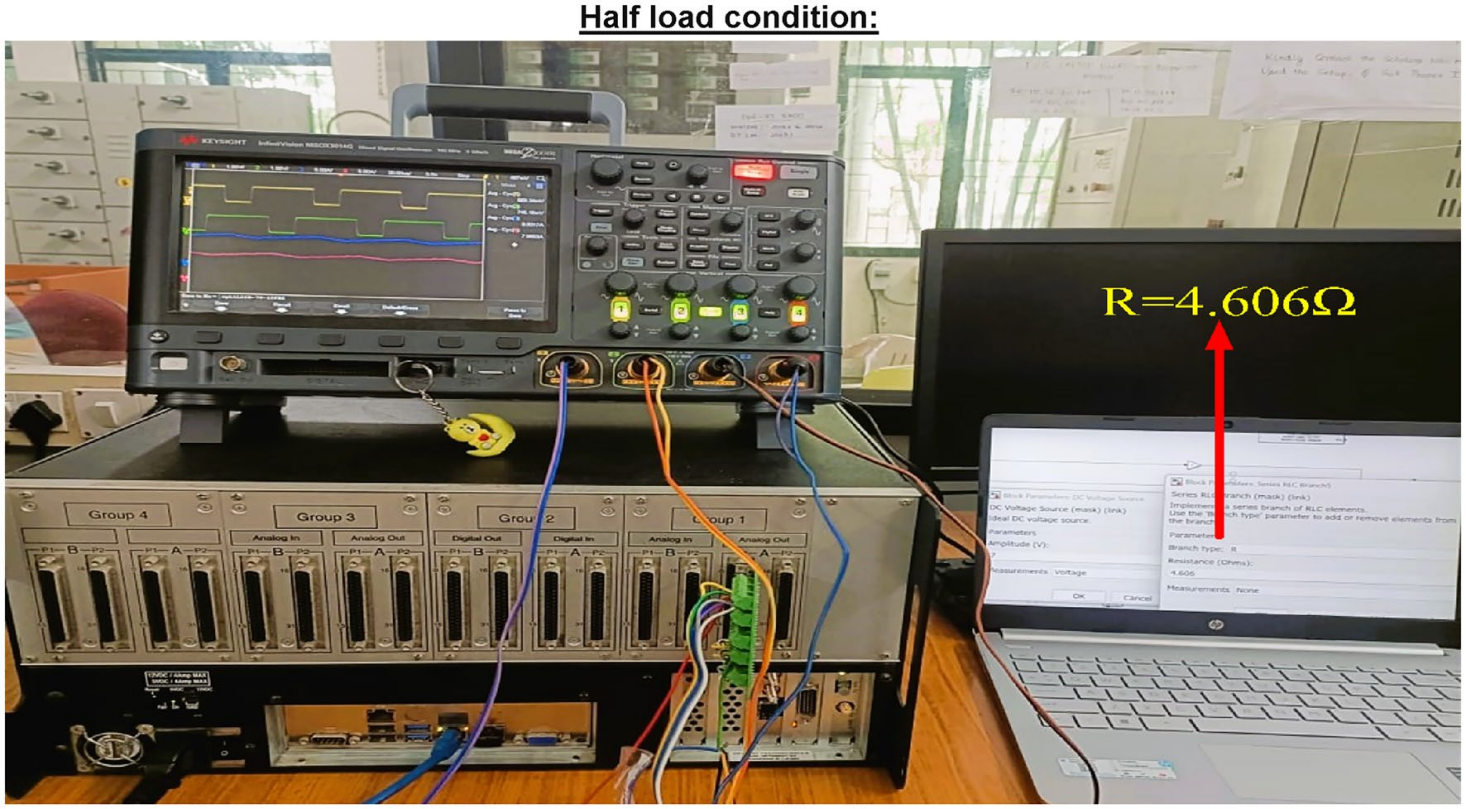
HIL experimental setup for the half load propoosed bual phase converter.

The optimal operation of fuel cells is attainable solely within the linear or ohmic region. Figure 18 signifies the full load condition, wherein upon the removal of the load, the stack voltage is anticipated to rise. To assess the functionality of the proposed DC to DC converter within the ohmic region, an experiment was conducted under half load conditions. Figure 27 shows the test bench setup for half load condition. The load value is doubled compared to full load condition. Hence current is reduced half and it indicates half load condition. We assumed stack voltage is raised to 7 V from 6 V while reducing the load.

**Figure 28:**
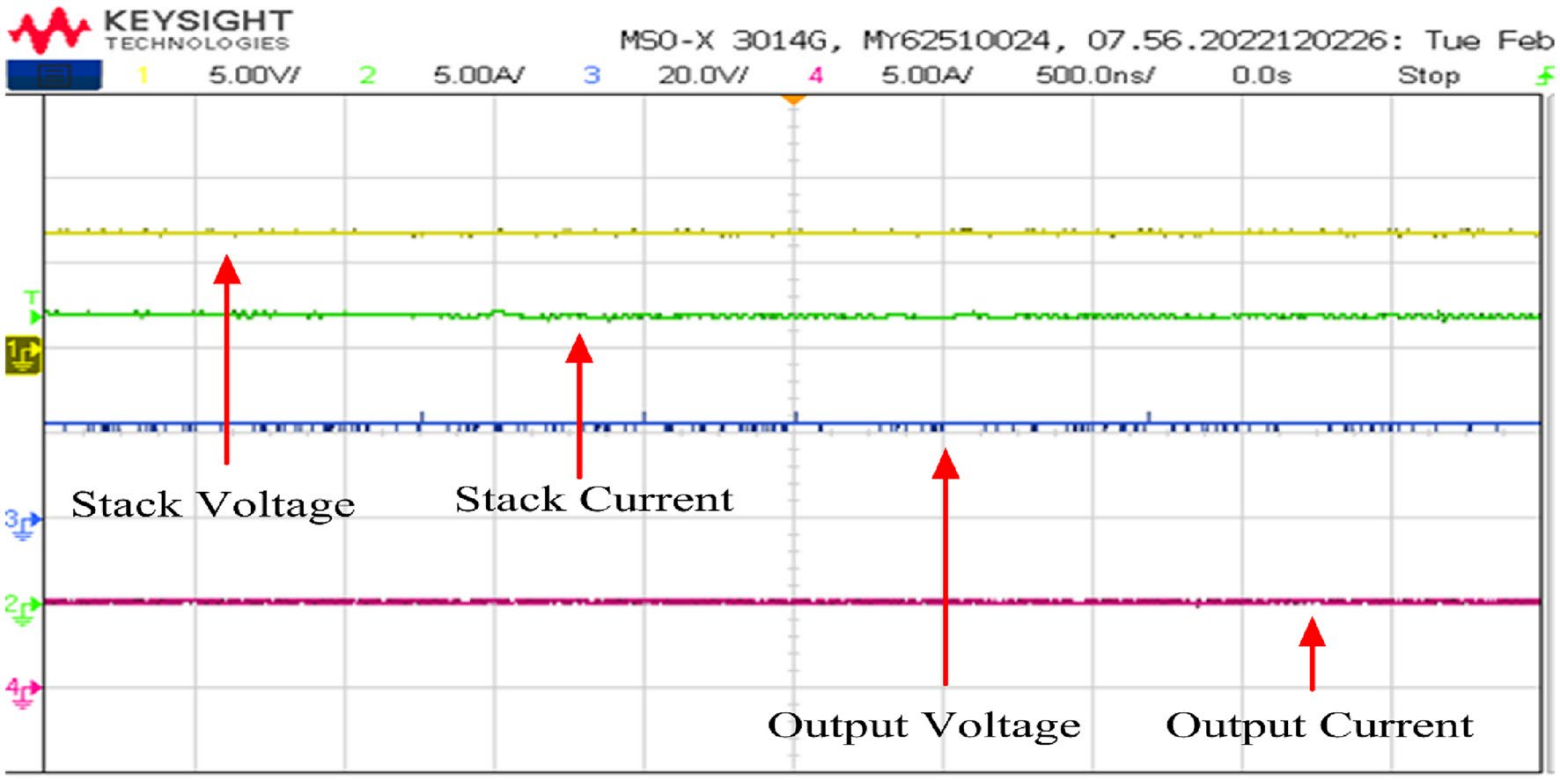
Stack voltage and stack current.

The proposed converter was operated at 50% of its peak power by modulating the load resistance from full load to half load (R = 4.606 Ω). Considering Fig. 28, the fuel cell stack voltage for a single cell under half load condition is approximately 0.7 V. Thus, we postulated that the stack voltage for the half load scenario would escalate from 6 to 7 V. Consequently, to uphold the consistent output voltage, the duty cycle was curtailed to 70.8%.

**Figure 29:**
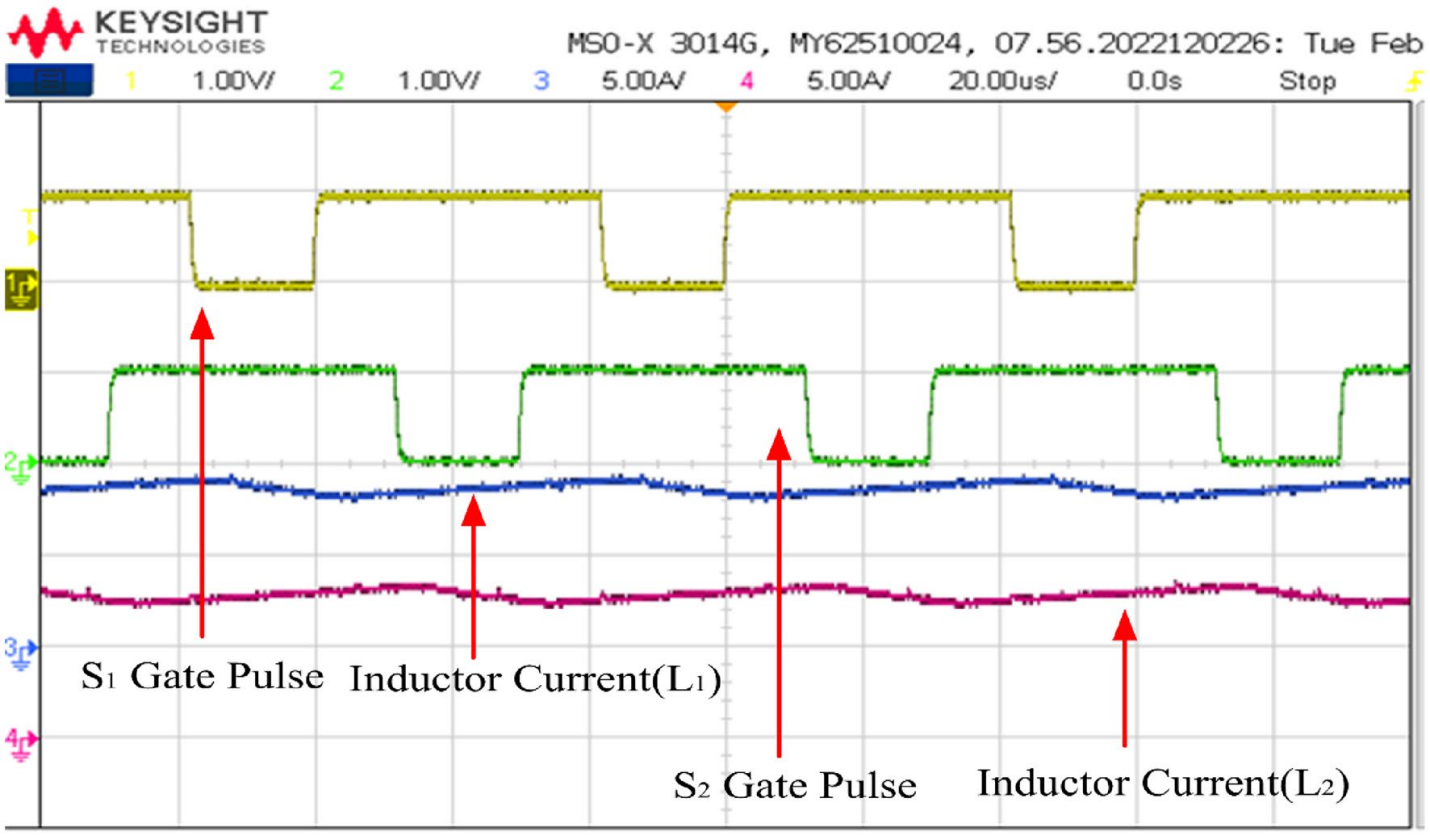
Inductor current waveforms with switching pulse.

The gate pulses and inductor current waveforms are depicted in Figs. 29 respectively. Figure 29 illustrates that the duty cycle is set at 70.8% while retaining the same switching frequency of 50 kHz. Furthermore, at half load condition, the input current is 17.9 A, and the two input inductors shares equally of 9 A.

To conduct a thorough economic analysis of power converters in fuel cell bicycle applications, several key parameters were considered: initial cost (including procurement and manufacturing), efficiency, lifespan, operational and maintenance costs, and total cost. These factors are crucial for assessing long-term economic viability. However, because the proposed converter was developed in a research lab, real-time economic analysis is challenging. Therefore, cost calculations focus on component costs, efficiency, and lifespan. Additionally, university research labs purchase components in limited quantities, leading to higher unit prices.

The number of components and device stress are compared and tableted in the introduction section. Table 6 presents a cost analysis of the proposed converter, comparing it with an existing suitable converter for the same application as documented in the literature. Table 6 shows that despite the proposed converter having additional inductors and capacitors for soft switching, its cost is lower than other converters. This cost efficiency is primarily due to interleaving the input port terminal.

**Table 6:** Cost comparison for the proposed converters with existing converters. Significant values are in bold.

Components details		Proposed converter	Double boost	Conventional boost
Inductors	Specifications	100 µH, 21A (RMS)	100µH,30A (RMS)	200µH,42A (RMS)
	Part number	CODACA-CPER3231-101MC	CPEX4141L-101MC	CODACA-CPCF3535-100 M
	Cost per unit	$16.26	$28.56	$15.55
	Quantity	2	2	20
	**Total cost**	**$32.52**	**$57.12**	**$311**
Switches	Specifications	30 V, 56 A	25 V,30 A	42A, 25 V
	Part number	IRLZ44N	Si7880ADP	BSC090N03LS G
	Cost per unit	$1.8	$1.2	$0.67
	Quantity	2	2	1
	**Total cost**	**$2.6**	**$2.4**	**$0.67**
Ultra-fast recovery diodes	Specifications	30 V, 56 A	25 V, 30 A	42A, 25 V
	Part number	MUR1560	BYV30W-600PT2	VS-HFA50PA60C-N3
	Cost per unit	$1.5	$1.8	$11.17
	Quantity	2	3	1
	**Total cost**	**$3**	**$5.4**	**$11.17**
Capacitors	Specifications	650µF, 30 V	521µF, 30 V	650µF, 30 V
	Part number	Nichicon UVR1V681MPD6	EEE-FT1V561AP	Vishay Beyschlag MAL213216681E3
	Cost per unit	$1.20	$1.27	$2.35
	Quantity	1	1	1
	**Total cost**	**$1.20**	**$1.27**	**$2.35**
Inductors (resonant)	Specifications	6 µH, 17A (RMS)	NIL	NIL
	Part number	CODACA-CSBX1809-6R8M		
	Cost per unit	$4.50		
	Quantity	2		
	**Total cost**	**$9**		
Capacitors (resonant)	Specifications	2.24 µF, 47 V	NIL	NIL
	Part number	WIMA FKP1 2.2/400/5		
	Cost per unit	$1		
	Quantity	2		
	**Total cost**	**$2**		
Heat sinks		**$3**	**$4**	**$2**
Controller ICs		**$10**	**$10**	**$10**
Manufacturing & assembly per unit (Includes PCB manufacturing, component soldering, and quality testing)		**$20**	**$25**	**$25**
Total components cost		$ 83.32	$ 105.19	$ 355.19

Assuming the fuel cell bicycle stack feeds the home DC grid for 8 h per day, with a fuel cell efficiency of 50%, an energy content of hydrogen at 33.33 kWh per kg, and an average hydrogen cost of $5 per kg^47,48^, the electricity generation cost from a 250 W fuel cell stack is approximately $0.30 per kWh. The proposed converter has an efficiency of 98%, and with 2920 operating hours per year, the annual energy cost for the converter is $4.38. Table 7 provides the complete economic analysis of the proposed converter with other existing converter.

**Table 7:** Economic analysis for the proposed converters with existing converters.

Parameter	Proposed converter	Double boost	Conventional boost
Components, manufacturing & assembly cost	$ 83.32	$ 105.19	$ 355.19
Operational costs (1 years)	$4.38	$43.8	$43.8
Maintenance cost (1 years)	$10	$12	$12
Total cost	$ 97.7	$ 160.99	$ 410.99

The study assumes a 10-year lifespan for the proposed dual-phase boost converter due to its 98% efficiency based on expected performance under optimal operating conditions, and regular maintenance, including inspections, cleaning, and occasional component replacements, is estimated to cost $10 to $50 per year^49^, in line with typical industry practices and manufacturer recommendations for similar power electronic devices. Hence the Operational cost for 10 years is $ 43.8 and maintenance cost for 10 years is $120 (approximately).

Similarly, for any hard-switching DC to DC converters, the efficiency typically ranges between 85 and 89%^50^. Assuming an efficiency of 89%, the same calculations were repeated for the other two existing converters, as presented in Table 7. From Tables 6 and 7, it is evident that the initial, operational, and maintenance costs for the proposed converter are more economical compared to the other existing converters.

## Conclusion

This research paper introduces a sustainable approach aimed at maximizing the utilization of energy from an electric bicycle stack during periods of inactivity, presenting a novel solution for powering home DC grids. The introduction of a dual-phase zero voltage switched DC to DC converter marks a significant advancement in boosting stack voltage and seamlessly integrating with 24 V DC home grid applications. Through rigorous simulation in the PSIM platform and real-world testing in HIL OPEL RT, the effectiveness and feasibility of the proposed converter have been validated.

The analysis delves into different scenarios of stack voltage (V_fc_ = 6 V, Full load & V_fc_ = 7 V, Half load) and fluctuations in load, stemming from the internal mechanisms of the fuel cell as load changes occur. The incorporation of the Zero Voltage Switching (ZVS) feature enables the utilization of a high switching frequency, enhancing efficiency to approximately 92.8%. However, further efficiency gains can be attained by replacing the existing diodes with switches. The division of the input stack current will lead to a decrease in the overall weight and volume of the magnetic components. This paper not only contributes to the burgeoning field of FCVs but also opens up avenues for sustainable energy harvesting and integration into domestic settings.

The primary objective of this paper revolves around examining the steady-state operation and analysis of the proposed dual phase DC/DC converter integrated with a bicycle fuel cell stack.. Experimental findings are presented to validate its steady-state performance. Future publications will delve into dynamic analysis and transient response assessments.

## Data Availability

The data used to support the findings of this study are included in the article.
